# Electrochemical Approach to Organonitrogen Compounds via C–N Coupling

**DOI:** 10.1002/advs.202513012

**Published:** 2025-09-19

**Authors:** Haifei Liu, Wenbo Wei, Qi‐Long Zhu

**Affiliations:** ^1^ School of Materials Science and Engineering Zhejiang Sci‐Tech University Hangzhou 310018 China; ^2^ Fujian Science & Technology Innovation Laboratory for Optoelectronic Information of China Fuzhou 350108 China; ^3^ State Key Laboratory of Structural Chemistry Fujian Institute of Research on the Structure of Matter Chinese Academy of Sciences (CAS) Fuzhou 350108 China; ^4^ University of Chinese Academy of Sciences Beijing 100049 China

**Keywords:** C–N coupling, electrocatalysis, electrocatalyst design, mechanistic understanding, organonitrogen compounds

## Abstract

The C–N bond is a cornerstone in the structures of many essential compounds, including amino acids, pharmaceuticals, agrochemicals, and natural products. Traditional methods for C–N coupling often rely on noble‐metal‐based catalysts and harsh reaction conditions, which limit their sustainability. In contrast, electrocatalytic C–N coupling has emerged as a more environmentally friendly alternative, offering mild reaction conditions, high selectivity, and compatibility with renewable energy integration. This review provides an overview of recent advances in the field of organonitrogen electrosynthesis, focusing on various nitrogen and carbon sources, their activation mechanisms, and the impact of key parameters such as applied potentials, pH values, and electrolyte composition. Various catalyst design strategies and electrochemical characterization techniques are also discussed. In addition, it examines different electrochemical cell configurations. Furthermore, this review highlights the electrosynthesis of key organonitrogen products such as urea, oximes, amino acids, amides, and amines, while discussing the current challenges and outlining future opportunities for advancing sustainable C–N bond formation.

## Graphical abstract

This review summarizes recent advances in electrocatalytic C–N coupling for organonitrogen electrosynthesis. It discusses innovative strategies for catalyst design and the electrosynthesis of diverse organonitrogen compounds, including urea, oximes, amino acids, amides, and amines. Additionally, it outlines future opportunities for sustainable C–N bond formation.

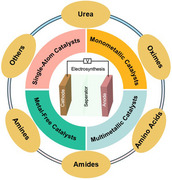

## 1 Introduction

The C–N bond is one of the most ubiquitous and essential chemical linkages in organonitrogen compounds, forming the backbones of amino acids, pharmaceuticals, agrochemicals, and numerous natural products.^[^ ^1^, ^2^, ^3^, ^4^, ^5^ ^]^ This bond is crucial not only in biological systems, where it plays a central role in protein structures and enzyme activity, but also in the development of therapeutic agents and industrial chemicals.^[^ ^6^, ^7^, ^8^ ^]^ For instance, in pharmaceuticals, the C–N bonds are integral to the structures of a wide array of drugs, including antibiotics, antivirals, and anticancer agents, which are designed to interact with biological targets in the body.^[^ ^9^ ^]^ In agrochemicals, C–N bonds are fundamental to the activity of herbicides, fungicides, and insecticides, helping to protect crops from pests and diseases.^[^ ^10^, ^11^ ^]^ Additionally, natural products such as alkaloids, which are derived from plants and animals, often contain C–N linkages that contribute to their bioactive properties.^[^ ^12^ ^]^ Given the widespread presence and importance of C–N bonds, their efficient formation through synthetic chemistry is key to advancing a broad range of fields, from biotechnology and medicine to agriculture and environmental science.^[^ ^13^, ^14^, ^15^ ^]^ Traditional C–N coupling methods often rely on redox reagents, noble‐metal‐based catalysts (e.g., platinum, gold, ruthenium, rhodium), and harsh reaction conditions (elevated temperature and pressure). While these methods are effective, they suffer from significant challenges in practical applications, such as high energy consumption, strict operation environments, poor reaction selectivity, and environmental pollution.^[^ ^16^, ^17^, ^18^, ^19^, ^20^, ^21^, ^22^ ^]^ Therefore, developing efficient and green C–N coupling methods is of great significance for modern chemical synthesis of organonitrogen compounds.

Electrocatalytic C–N coupling reactions have emerged as a promising approach to addressing energy and environmental challenges. By electrochemically activating various nitrogen and carbon sources, include those from the atmosphere or environmental pollutants, valuable organonitrogen chemicals can be synthesized like urea, oximes, amino acids, amides, amines, etc.^[^ ^23^, ^24^, ^25^, ^26^, ^27^, ^28^, ^29^, ^30^, ^31^ ^]^ Compared to traditional methods, electrocatalytic C–N coupling reactions offer several key advantages.^[^ ^32^, ^33^, ^34^, ^35^, ^36^, ^37^ ^]^ First, electrochemical reactions are driven directly by electricity, eliminating the need for excessive redox reagents, elevated temperature and high‐pressure conditions typically required by conventional catalytic approaches. This results in milder reaction conditions and higher energy efficiency. Second, electrochemical approach provides good selectivity, particularly in complex substrate transformations. By precisely tuning the current and potential, reaction pathways can be effectively controlled, minimizing side reactions. Most importantly, electrochemical synthesis is safer, more flexible, and can be integrated with renewable energy sources such as wind, tidal, and solar power. These reactions can operate under ambient temperature and pressure, making them a sustainable and environmentally friendly alternative to traditional chemical processes.

Although significant advances have been made in electrocatalytic C–N coupling reactions, the field remains largely in the exploratory and experimental stages, especially regarding mechanistic understanding. The complexity of multistep reactions and the diversity of reactive intermediates present major challenges in fully elucidating the reaction mechanisms. This review offers a comprehensive overview of current progress in electrocatalytic C–N coupling, starting with an examination of various nitrogen and carbon sources (such as N_2_, NH_3_, NO_x_, amines, CO_2_, alcohols, etc.) and their respective activation mechanisms in electrochemical systems. The review highlights the differences in reactivity among these nitrogen species and their roles in C–N bond formation. Key factors such as applied potentials, pH values, and electrolyte composition are discussed in relation to their influence on product distribution and catalytic performance in various electrochemical systems.^[^ ^38^, ^39^, ^40^ ^]^ Additionally, this review summarizes common electrochemical techniques used to study C–N coupling reactions, such as cyclic voltammetry, linear sweep voltammetry, chronoamperometry, and electrochemical impedance spectroscopy.^[^ ^41^, ^42^, ^43^ ^]^ These methods provide valuable insights into reaction kinetics, intermediate species, and product quantification, thus offering essential support for optimizing reaction conditions and enhancing selectivity. The review also introduces three promising electrochemical cell types for C–N bond formation: H‐type electrolytic cells, flow cells, and membrane electrode assembly (MEA) electrolyzers.^[^ ^41^ ^]^ Furthermore, the review delves into the design strategies for electrocatalysts, focusing on both transition metal catalysts (such as copper, nickel, iron, palladium, etc.) and metal‐free catalysts (such as carbons doped with nitrogen, boron, and fluorine, etc.) in C–N coupling reactions.^[^ ^44^, ^45^, ^46^, ^47^, ^48^, ^49^, ^50^, ^51^ ^]^ Subsequently, we examine the synthesis pathways of a range of organonitrogen compounds, including urea, oximes, amino acids, amides, and amines, etc. Particular attention is given to how various catalysts facilitate these transformations through distinct catalytic mechanisms, with an emphasis on the influence of catalyst design and reaction conditions in improving product selectivity and overall efficiency. Finally, this review highlights the major challenges and future opportunities in this field, including issues related to catalyst stability, product selectivity, substrate scope, and mechanistic complexity. Although electrocatalytic C–N coupling reactions have been the subject of several comprehensive reviews in the literature, this work offers a fresh perspective by addressing emerging trends and aspects that have received less attention in prior studies. With continuous advancements in catalyst design, process optimization, and mechanistic studies, electrocatalytic C–N coupling reactions are poised to expand their application potential, especially in pharmaceutical synthesis, environmental remediation, and sustainable energy conversion.^[^ ^52^, ^53^, ^54^, ^55^, ^56^ ^]^

## 2 Fundamental Principles of Electrochemical C–N Coupling Reactions

### 2.1 Different Nitrogen and Carbon Sources as Reaction Feedstocks

**Figure 1:**
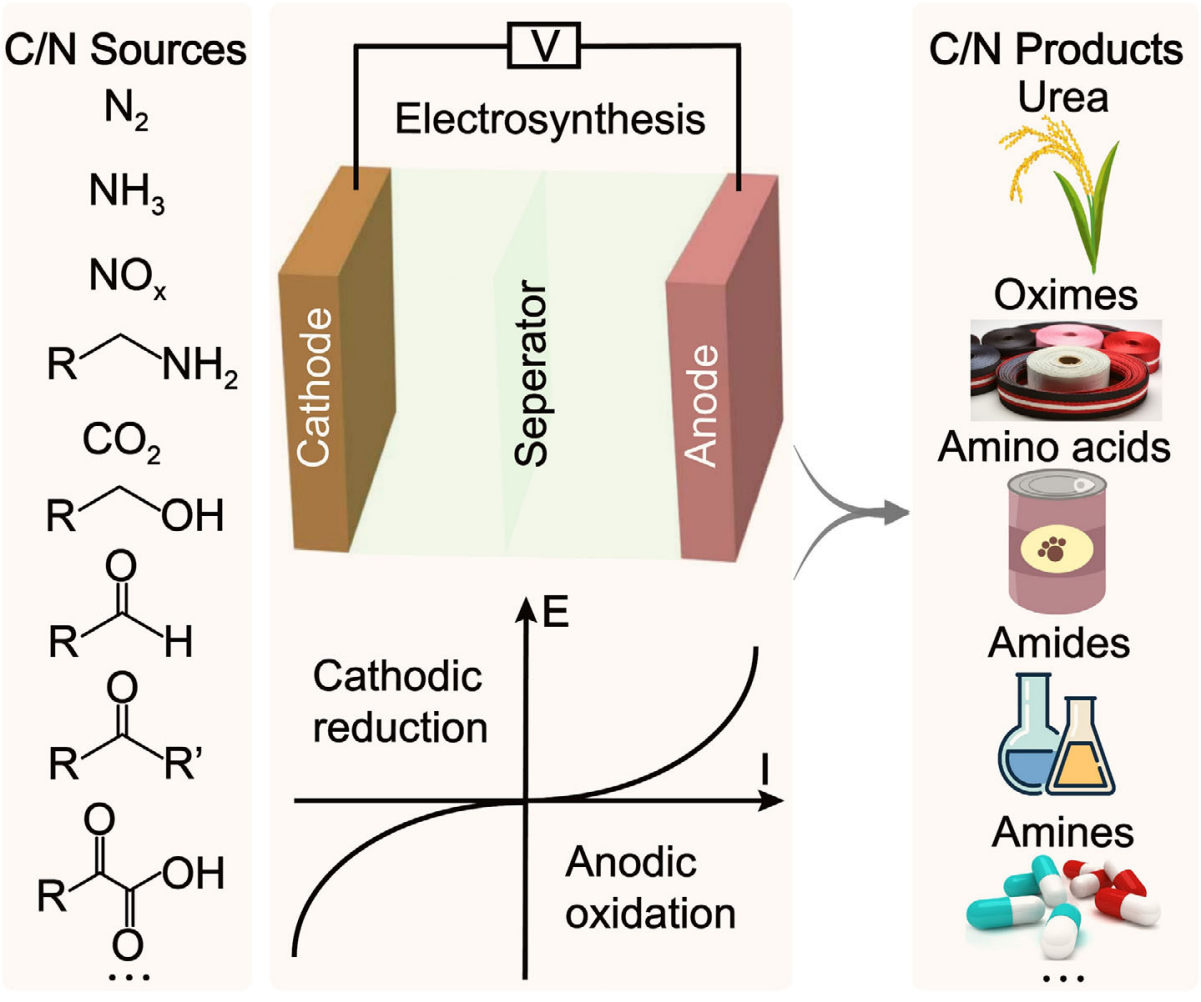
Electrocatalytic synthesis of various organonitrogen compounds via different nitrogen and carbon sources.

Electrochemical reactions between various nitrogen sources (such as N_2_, NH_3_, NO_x_, amines, etc.) and a wide range of carbon sources (such as CO_2_, alcohols, aldehydes, ketones, organic acids, etc.) enable the synthesis of diverse organonitrogen compounds (**Figure** **1**), including urea, oximes, amino acids, amides, amines, etc.^4^ These reactions not only play a crucial role in organic synthesis but also hold great promise in nitrogen and carbon cycle optimization and the resource utilization of wastes. Using N_2_ as a nitrogen source typically involves its activation and reduction, most notably through the electrochemical nitrogen reduction reaction (NRR), which cleaves the inert N≡N triple bond to generate NH_3_. Subsequently, NH_3_ can undergo coupling reactions with CO_2_ to produce nitrogen‐containing products like urea.^[^ ^57^, ^58^ ^]^ Although the direct activation of N_2_ requires considerable energy input, its abundance and environmental friendliness make it a highly attractive nitrogen source in green chemistry. NH_3_, with its relatively high reactivity, is commonly used as a conventional nitrogen feedstock.^[^ ^59^ ^]^ It can react with a variety of carbon‐based compounds (such as aldehydes, ketones, alkenes, organic acids, and acid anhydrides) to produce oximes, amines, amides, and amino acids. For instance, NH_3_ reacts with aldehydes or ketones to form oximes and with acids or anhydrides to form amides, and under specific conditions, it can undergo amination with organic acids to yield amino acids. NO_x_, including NO, NO_2_, NO_2_^−^, and NO_3_^−^, represent another class of widely available and recyclable nitrogen sources, particularly suited for electrochemical conversion.^[^ ^60^, ^61^ ^]^ Under electrocatalytic conditions, NO_x_ can be reduced to a series of reactive intermediates such as NH_2_OH or NH_3_, which can then couple with CO_2_ or carbon‐containing organic molecules (such as aldehydes or ketones) through C–N coupling reactions to produce high‐value compounds like urea, amines, and oximes. This not only offers a sustainable solution for the green conversion of nitrogen‐containing exhaust gases or water but also presents a novel strategy for the integrated utilization of carbon‐based greenhouse gases like CO_2_. In addition, amines, owing to their inherent nucleophilicity and structural diversity, exhibit high selectivity and controllability in C–N coupling reactions.^[^ ^62^ ^]^ The in situ generated carbonyl intermediates (such as *CHO or *CO) can undergo nucleophilic addition with primary or secondary amines to form structural units such as amides, methylamines, and dimethylamines.

### 2.2 Reaction Influencing Factors

The efficiency and selectivity of electrocatalytic C–N coupling reactions are influenced by a range of interconnected factors, including the properties of the electrocatalysts, applied potentials, pH values, electrolyte composition, and reactant adsorption behaviors.^[^ ^38^, ^39^, ^40^ ^]^ The electrocatalysts must offer active sites capable of adsorbing and activating both carbon and nitrogen sources. Achieving the optimal binding strength is crucial. If the binding is too weak, reactants may not remain on the catalyst surface long enough for the reaction to occur, while if it is too strong, product release may be hindered. Advanced catalysts often employ strategies like dual‐site adsorption or tuning the electronic structures. Single‐atom catalysts provide highly active sites that stabilize intermediates, while bimetallic catalysts take advantage of synergistic interactions between metal species to enhance selectivity. The applied potentials and pH values play a key role in determining the reaction pathways and minimizing side reactions such as hydrogen evolution reaction (HER). The pH values also affect the protonation state of nitrogen intermediates, which in turn influences the efficiency of C–N coupling. Electrolyte composition affects ion transport and catalyst stability, with specific ions or additives modulating the overall catalytic process. In summary, optimizing the efficiency and selectivity of C–N coupling reactions requires careful design of the catalysts, as well as precise control over applied potentials, pH values, and electrolyte composition. Innovations in catalyst design, such as single‐atom catalysts and bimetallic systems, offer promising strategies to enhance performance.

### 2.3 Testing Methods for Electrochemical C–N Coupling

The characterization methods for electrochemical C–N coupling are essential for assessing catalyst performance in terms of activity, selectivity, durability, and reaction kinetics. Frequently employed electrochemical techniques include cyclic voltammetry (CV), linear sweep voltammetry (LSV), chronoamperometry (CA), and electrochemical impedance spectroscopy (EIS).^[^ ^41^, ^42^, ^43^ ^]^ CV is used to probe the redox characteristics of catalysts and the reversibility of surface‐active sites. It also enables estimation of the electrochemically active surface area (ECSA) through double‐layer capacitance analysis, facilitating normalization of current densities. LSV provides current‐potential profiles and is commonly applied to evaluate the catalytic response of materials toward C–N coupling. CA involves monitoring current over time at a fixed potential, offering insights into the operational stability of catalysts. Coupled with quantitative product detection methods such as gas chromatography (GC) or high‐performance liquid chromatography (HPLC), CA data can be used to calculate Faradaic efficiency (FE) and product yield. EIS provides further understanding of the reactions by quantifying charge transfer resistance and diffusion behaviors, shedding light on the kinetics and interfacial dynamics. Together, these techniques provide a comprehensive toolkit for elucidating the mechanisms and optimizing the efficiency of C–N bond formation in the electrocatalytic systems.

### 2.4 Electrochemical Cell Configurations

**Figure 2:**
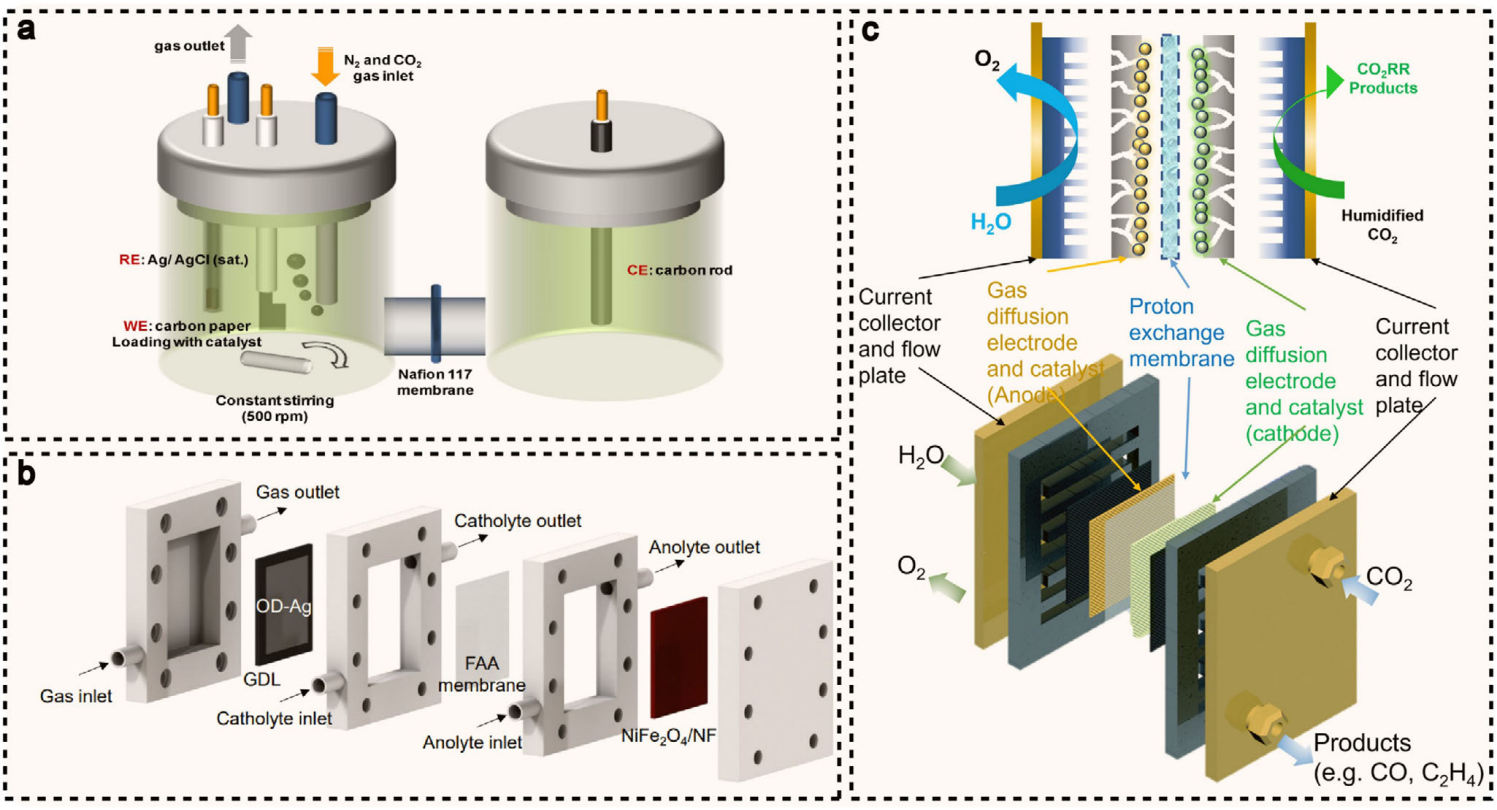
a). A representation showcasing the H‐type cell layout tailored for urea synthesis. Reproduced with permission.^[^ ^63^ ^]^ Copyright 2020, Springer Nature. b) The schematic portrays a dual‐compartment flow cell dedicated to the electroreduction of propylene oxide. Reproduced with permission.^[^ ^64^ ^]^ Copyright 2023, Springer Nature. c) A schematic detailing the MEA cell utilized for the electrocatalysis of CO_2_. Reproduced with permission.^[^ ^65^ ^]^ Copyright 2022, Elsevier.

Electrochemical C–N coupling reactions typically require carefully designed cell configurations to optimize reaction efficiency, selectivity, and product distribution. The choice of electrochemical cell is crucial, as it directly influences factors such as mass transport, reaction kinetics, and the ability to control the reaction environment. Common cell configurations for electrochemical C–N coupling reactions include H‐type cell, flow cell, and membrane electrode assembly (MEA) cell, each with distinct advantages and challenges that make them suitable for different scales and types of reactions. The H‐type cell is the most commonly used laboratory‐scale cell configuration. It consists of two reaction chambers, namely the cathode chamber and the anode chamber, separated by an ion exchange membrane (**Figure** **2**a).^[^ ^63^ ^]^ Although the H‐type cell is relatively easy to set up and use for fundamental studies, their scalability is limited by issues related to mass transport and product separation, making them less suitable for large‐scale industrial applications. The flow cell is an advanced electrochemical reactor engineered for continuous operation, with an architecture specifically optimized to achieve enhanced mass transport, efficient gas/liquid management, and stable electrocatalytic performance. When processing gaseous reactants, the system can incorporate a gas diffusion electrode (GDE) comprising a gas diffusion layer (GDL) for reactant distribution and a catalyst layer (CL) for electrochemical conversion, which is essential for maintaining efficient three‐phase (gas/liquid/solid) interfacial reactions (Figure 2b).^[^ ^64^ ^]^ The advantage of the flow cell lies in its ability to continuously supply reactants and efficiently remove products, overcoming the limitations of H‐type cell in gas‐liquid contact. However, it faces challenges such as higher cell resistance, GDE clogging, and precipitated salt accumulation, which limit its large‐scale application. Despite these issues, the flow cell still holds great potential for electrochemical C–N coupling reactions, particularly in the generation of reaction products at high current densities. The MEA cell is an efficient electrochemical device widely used in proton exchange membrane fuel cells and electrolyzers. It consists of a proton exchange membrane, catalytic layer, and gas diffusion layer (GDL) (Figure 2c),^[^ ^65^ ^]^ allowing humidified gases to replace liquid electrolytes, avoiding clogging issues and reducing resistance. The MEA cell demonstrates significant promise for improving reaction efficiency at high current densities and simplifying product collection. However, some technical challenges remain, particularly in long‐term durability and large‐scale production. Despite these challenges, it still show considerable potential for advancing a wide range of electrochemical processes, including C–N coupling reactions. The key parameters for each reactor type in C–N coupling applications are shown in **Table** **1**.

**Table 1:** A summary of the key parameters for each reactor type in C–N coupling applications.

Cell Configurations	Max current density [A cm^−2^]	FE	Yield [mol h^−1^ m^−2^]	Durability	Refs.
H‐cell	0.1–0.5	50%‐70%	1–5	Medium	[63]
Flow cell	0.5–1.0	60%‐80%	5–10	High	[64]
MEA cell	0.1–1.0	70%‐85%	10–50	High	[65]

Note: “FE” stands for Faradaic Efficiency, “MEA” stands for membrane electrode assembly.

## 3 Catalyst Design for Electrochemical C–N Coupling

### 3.1 Single‐Atom Catalysts

**Figure 3:**
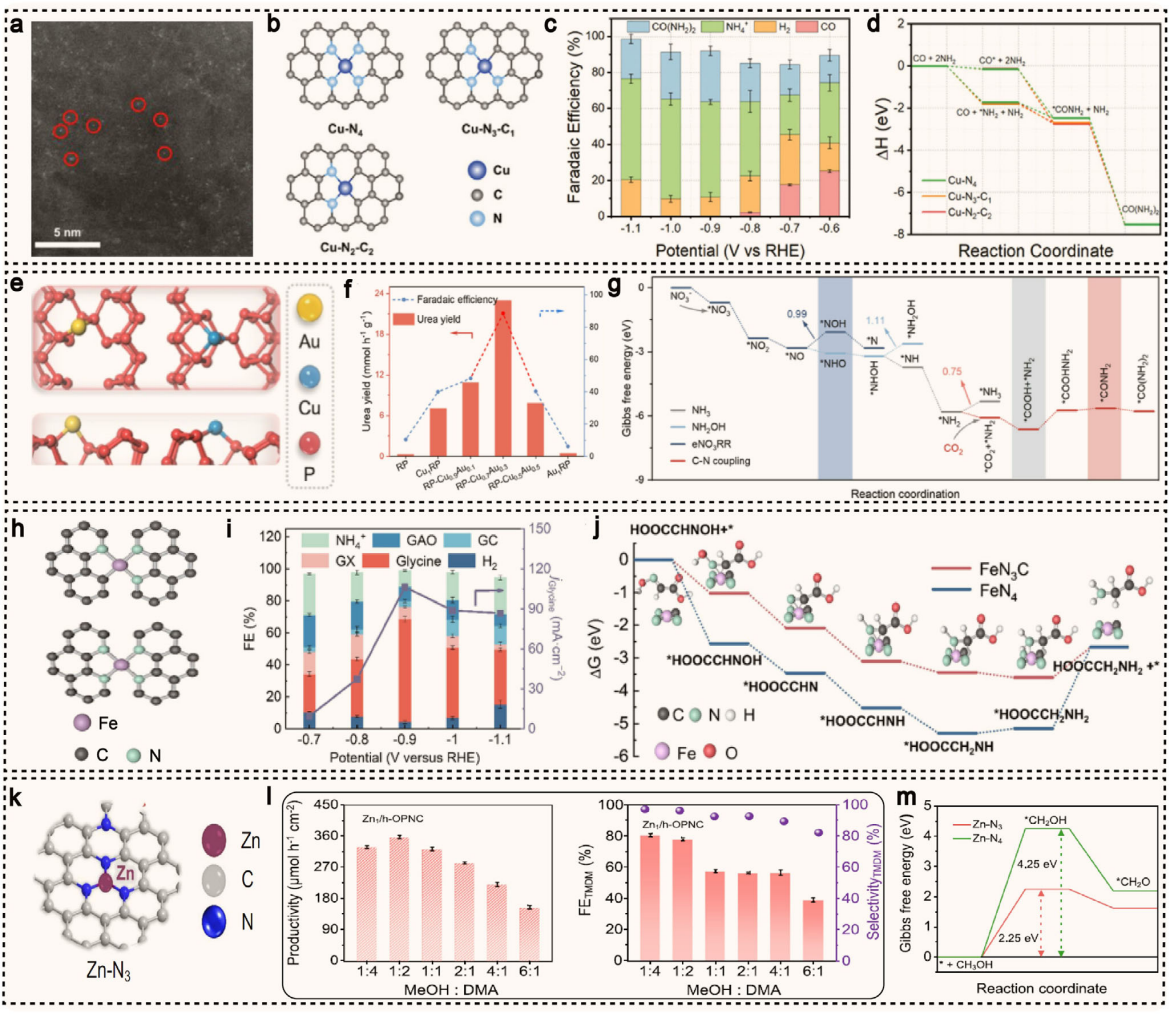
a) HAADF‐STEM imaging of Cu‐GS‐800 (some single atoms are circled in red). b) Graphical illustrations of the modelled Cu‐N‐C sites. c) FEs in the H‐cell system using Cu‐GS‐800. d) The reaction mechanism for urea synthesis through the simultaneous electrochemical CO_2_RR and NO_3_RR. Reproduced with permission.^[^ ^77^ ^]^ Copyright 2022, Wiley‐VCH. e) Graphical illustrations of Cu and Au single atoms on the red phosphorus. f) Electrocatalytic performance screening of RP‐Cu_x_Au_1−x_ (x = 0, 0.5, 0.7, 0.9, 1) composites for urea electrosynthesis. g) Gibbs free energy profiles for the co‐reduction of NO_3_^−^ and CO_2_, with different activation and coupling stages of NO_3_^−^ and CO_2_ marked by distinct colors. Reproduced with permission.^[^ ^78^ ^]^ Copyright 2025, American Chemical Society. h) Graphical illustrations of the modelled Fe‐N‐C sites. i) Plot of *j*_Glycine_ and FE versus potential in 0.5 M OA+0.5 M NaNO_3_ over Fe‐N‐C‐700. j) Free energy change of glyoxylic acid oxime (GAO) reduction to glycine over FeN_3_C and FeN_4_. Reproduced with permission.^[^ ^79^ ^]^ Copyright 2024, American Chemical Society. k) Graphical illustrations of the modelled Zn‐N_3_ site. l) Productivity and FE_TMDM_ of Zn_1_/h‐OPNC at 0.8 V in 0.7 M K_2_CO_3_ with different concentration ratios of MeOH and DMA. m) Gibbs free energy for the formation of *CH_2_O over the Zn‐N_3_ and Zn‐N_4_ model configurations. Reproduced with permission.^[^ ^80^ ^]^ Copyright 2025, Springer Nature.

Single‐atom catalysts (SACs) offer remarkable advantages in electrocatalytic C–N coupling reactions, owing to their atomically dispersed metal sites and highly tunable electronic structures. Compared to traditional noble‐metal catalysts, SACs offer several distinct advantages, most notably their ability to fully utilize metal atoms as active sites, leading to significantly improved atomic efficiency. The highly dispersed nature of SACs ensures that each metal atom participates in the catalytic process, often resulting in enhanced catalytic activity and selectivity.^[^ ^66^, ^67^, ^68^, ^69^ ^]^ Moreover, SACs typically demonstrate superior stability due to reduced sintering and aggregation compared to their bulk counterparts, making them more durable during long‐term catalytic cycles. However, despite these advantages, SACs face challenges in synthesis and scalability, which can complicate their practical application. In SACs, individual metal atoms are anchored onto the support surface, achieving maximum atomic utilization while generating uniform and well‐defined active sites.^[^ ^70^, ^71^, ^72^ ^]^ This atomic precision allows for fine‐tuning of reaction pathways and improved catalytic performance. Typically, these isolated metal centers are coordinated with non‐metallic atoms such as nitrogen, carbon, or oxygen (e.g., M–N_4_ and M‐N_3_C configurations), forming stable and catalytically active structures.^[^ ^73^, ^74^ ^]^ In SACs with M‐N_4_ coordination, the metal atom is coordinated to four nitrogen atoms in a square planar geometry. This structure tends to stabilize *NH_2_ intermediates through favorable binding interactions with the nitrogen atoms, which can facilitate the hydrogenation step essential for C–N bond formation. The increased electronic density at the metal center, induced by the N_4_ coordination, can also favor the activation of nitrogen‐based intermediates, making it easier for the catalyst to facilitate C–N coupling. On the other hand, in M‐N_3_C coordination environments, the metal is coordinated to three nitrogen atoms and one carbon atom. This configuration can influence the interaction with *CO intermediates. The presence of a carbon ligand can induce electronic effects that favor the adsorption of *CO intermediates, enhancing their participation in C–N coupling reactions. The carbon atom acts as an electron‐donating group, stabilizing the *CO intermediate and facilitating its subsequent reaction with *NH_2_ to form the C–N bond. By adjusting the local coordination environments, SACs can optimize the adsorption and activation of crucial intermediates, thereby facilitating efficient C–N bond formation. In addition, SACs exhibit excellent selectivity by suppressing competing side reactions like the HER,^[^ ^75^, ^76^ ^]^ and they maintain robust catalytic stability across a wide potential range. These combined features position SACs as promising platforms for the electrocatalytic synthesis of value‐added organonitrogen compounds, including urea, amino acids, and anti‐tumor drugs. For instance, Amal et al. mixed a solution of copper salts, glucose, and dicyandiamide with NaCl, followed by freeze‐drying and subsequent annealing under an Ar atmosphere, successfully constructing a Cu–N_x_–C_x_ coordination structure (**Figure** **3**a).^[^ ^77^ ^]^ As the annealing temperature increased from 800 to 1000 °C, the coordination environment of the copper atoms gradually transforms from Cu–N_4_ sites to Cu–N_3_–C_1_ and Cu–N_2_–C_2_ sites (Figure 3b). Using CO_2_‐saturated KHCO_3_ as the electrolyte, the Cu–N_4_ sites exhibit a FE for CO production as high as 59% at −0.8 V (vs RHE), significantly outperforming the Cu–N_3_–C_1_ and Cu–N_2_–C_2_ configurations (Figure 3c). Density functional theory (DFT) calculations reveal that the energy barriers for C–N bond formation from *CO and *NH_2_ intermediates were comparable across the different coordination structures (Figure 3d). The key to enhancing the carbon dioxide reduction reaction (CO_2_RR) performance lies in the undercoordinated nitrogen atoms surrounding the isolated Cu centers, which strongly interact with CO_2_, thereby significantly promoting the overall reaction. As a result, Cu–N_4_ sites exhibit superior catalytic activity for CO_2_ reduction, leading to higher urea yields. Besides, Yang et al. developed a dual single‐atom catalyst supported on red phosphorus (RP) (Figure 3e), referred to as RP‐AuCu.^[^ ^78^ ^]^ By leveraging the synergistic interaction between the Au and Cu single atoms, the catalyst enables the electrocatalytic co‐reduction of CO_2_ and NO_3_^−^ in an aqueous medium. At an applied potential of −0.6 V (vs RHE), RP‐AuCu achieves a urea yield of 22.9 mmol g_cat._^−1^ h^−1^, with a remarkable FE of 88.5% at −0.5 V (vs RHE) (Figure 3f). Compared to monometallic SACs (Au_1_/RP and Cu_1_/RP), RP‐AuCu demonstrates significant performance enhancement of 3 to 80 times. Computational studies reveal that all the steps in the reduction of *NO_3_^−^ to *NH_2_ at the Cu site are exothermic, making the formation of *NH_2_ thermodynamically favorable (Figure 3g). During the urea synthesis process, Au sites preferentially facilitate the CO_2_RR and promote C–N coupling on the carbon side, whereas Cu sites enhance the nitrate reduction reaction (NO_3_RR).

In addition to conventional urea synthesis, the electrocatalytic synthesis of amino acid using SACs also holds significant importance. Han et al. synthesized two types of iron‐based SACs with different coordination structures by tuning the pyrolysis temperature.^[^ ^79^ ^]^ In Fe‐N‐C‐600 and Fe‐N‐C‐700, the Fe(III) centers adopt a four‐coordinate structure (FeN_3_C) composed of three nitrogen atoms and one carbon atom, while Fe‐N‐C‐800 exhibits a typical FeN_4_ coordination configuration (Figure 3h). Using an electrolyte composed of 0.5 M oxalic acid (OA) and 0.5 M NaNO_3_, Fe‐N‐C‐700 enables the co‐reduction of OA and NO_3_^−^. The FE for glycine production (FE_Glycine_) shows a volcano‐type trend with respect to the applied potential, reaching a maximum of 64.2% at −0.9 V (vs RHE) (Figure 3i). DFT calculations reveal that the FeN_3_C structure in Fe‐N‐C‐700, in synergy with neighboring pyrrolic nitrogen atoms, facilitates the reduction of OA to glyoxylic acid, a crucial step for the subsequent formation of glyoxime and glycine. Moreover, the FeN_3_C coordination environment significantly lowers the energy barrier for the formation of the *HOOCCH_2_NH_2_ intermediate, thereby accelerating the conversion from glyoxime to glycine (Figure 3j).

Recently, Zhu et al. employed polystyrene spheres as the template to successfully synthesize ZIF‐8 single crystals with a 3D interconnected ordered macroporous structure (OM‐ZIF‐8).^[^ ^80^ ^]^ The OM‐ZIF‐8 precursor was then mixed with dicyandiamide (DCDA) and pyrolyzed in an argon atmosphere at 1000 °C, yielding the Zn_1_/h‐OPNC material. This material features accessible under‐coordinated Zn‐N_3_ sites (Figure 3k), providing ideal active sites for subsequent electrocatalytic reactions. Zn_1_/h‐OPNC demonstrates outstanding activity and selectivity in electrocatalytic N–C–N coupling reactions. Notably, as a representative N–C–N bonded compound, TMDM (N,N,N″,N″‐tetramethyldiaminomethane) can be efficiently synthesized, exhibiting ultra‐high productivity (357 µmol h^−1^ cm^−2^), nearly 100% selectivity (96%), and a high FE of 77% at 0.8 V (Figure 3l). Moreover, the researchers successfully developed an electro‐thermal cascade strategy, using TMDM as a mediator for the synthesis of high‐value organonitrogen compounds with (dimethylamino)methyl substituent. This approach includes the gram‐scale synthesis of the anticancer drug topotecan hydrochloride, with a yield of 95%. DFT calculations reveal that the under‐coordinated Zn‐N_3_ sites in Zn_1_/h‐OPNC facilitate methanol oxidation, producing the key intermediate *CH_2_O, which in turn promotes the subsequent nucleophilic addition reaction with amines (Figure 3m). Thanks to the uniform distribution of active sites at the atomic scale and the tunable electronic structure, SACs exhibit unique advantages in promoting C–N bond formation, as well as enhancing reaction selectivity and efficiency.^[^ ^81^, ^82^ ^]^ Therefore, the development of efficient SACs for the targeted synthesis of complex organonitrogen molecules, such as amino acids and anti‐tumor drugs, holds great promise for making a profound impact on fields such as medicine, agriculture, and materials science.

### 3.2 Monometallic Catalysts

**Figure 4:**
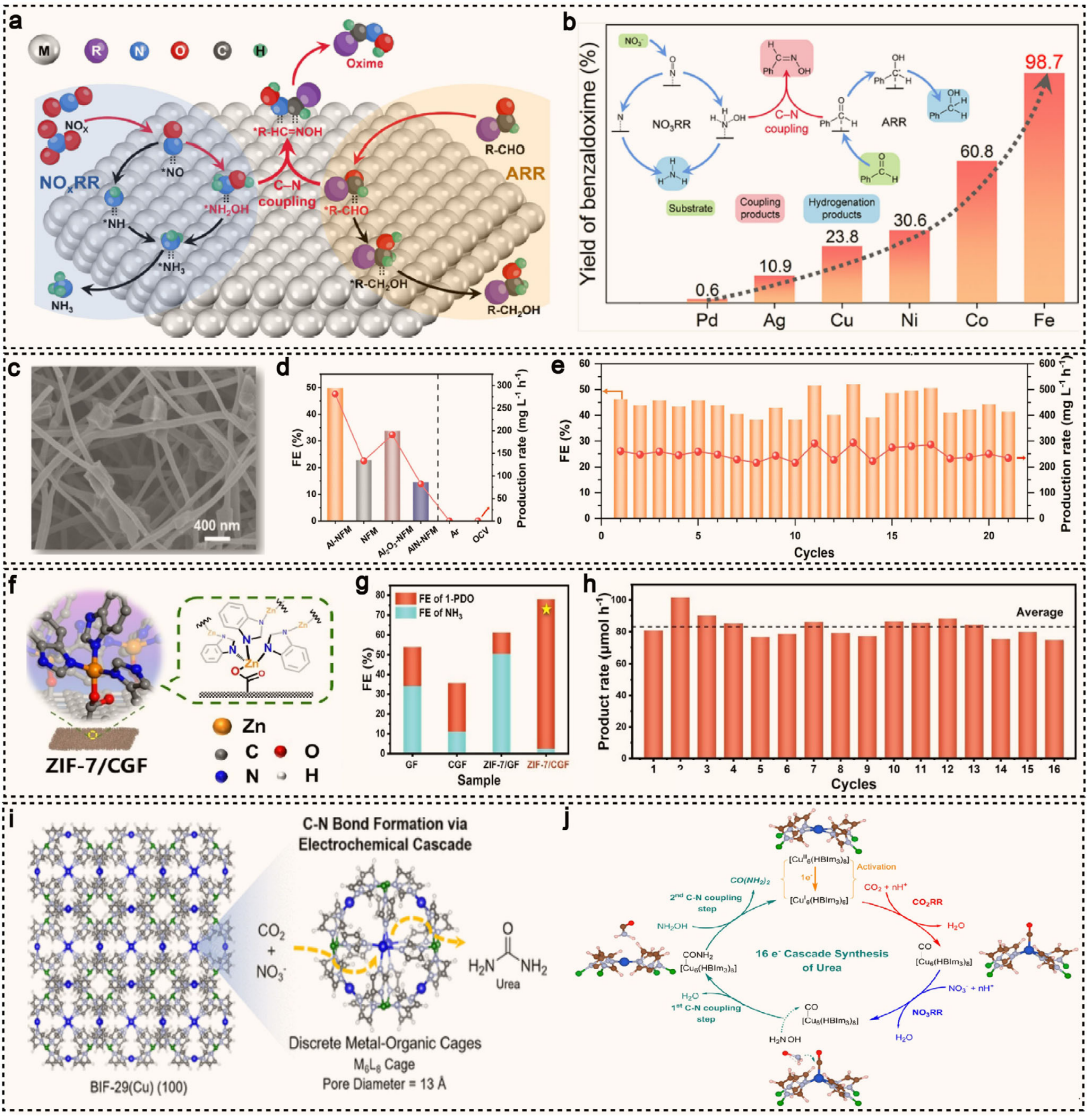
a) Schematic diagram illustrating the direct electrosynthesis of oxime via the electrochemical co‐reduction of NO_x_ and R‐CHO. b) Graphical illustrations showing that the Fe catalyst outperforms other metal catalysts in electrochemical co‐reduction. Reproduced with permission.^[^ ^46^ ^]^ Copyright 2024, American Chemical Society. c) SEM of Al‐NFM. d) The FE and production rate of Al‐NMF and compared specimens and control experimental condition. e) stability test of Al‐NFM catalyst with corresponding FE and production rate. Reproduced with permission.^[^ ^90^ ^]^ Copyright 2023, Wiley‐VCH. f) Graphical illustration showing synthetic ZIF‐7/CGF. g) The FE and yield of electrosynthesis 1‐PDO and NH_3_ over GF, CGF, ZIF‐7/GF and ZIF‐7/CGF. h) The long‐term stability test for 1‐PDO production on ZIF‐7/CGF. Reproduced with permission.^[^ ^91^ ^]^ Copyright 2025, Wiley‐VCH. i) Schematic diagram illustrating the electrochemical reduction of CO_2_RR and NO_3_RR using BIF‐29(Cu) for urea synthesis. j) Proposed mechanism for urea production from CO_2_ and NO_3_^−^ within the BIF‐29(Cu) cage. Reproduced with permission.^[^ ^92^ ^]^ Copyright 2023, American Chemical Society.

In recent years, significant progress has been made in the development of monometallic catalysts for electrocatalytic C–N coupling reactions. Researchers have discovered that by precisely tuning the type of metal (such as Cu, Ni, or Fe) and its electronic structure, the selectivity and efficiency of C–N bond formation can be substantially enhanced. For instance, copper‐based catalysts have been widely employed in the electrocatalytic synthesis of urea, amides and amino acids,^[^ ^83^, ^84^, ^85^ ^]^ owing to their strong capabilities in reducing NO_x_ and activating carbon sources. In addition, strategies such as constructing atomically dispersed metal sites, controlling crystal facets, and introducing surface defects have been adopted to further boost catalytic activity and stability.^[^ ^86^, ^87^ ^]^ These advancements not only drive the sustainable development of electrocatalytic C–N coupling technologies but also open new avenues for the green synthesis of nitrogen‐containing organic compounds. Notably, Zou et al. recently pioneered an aqueous electrochemical reductive C–N coupling system for the direct electrosynthesis of oximes from nitrogen oxides (NO_x_) and aldehydes (R‐CHO) (**Figure** **4**a).^[^ ^46^ ^]^ By integrating experimental and theoretical methods, they identified iron (Fe) as the optimal catalyst, capable of effectively enriching key reaction intermediates and facilitating their C–N coupling, thereby enabling highly efficient oxime production. The Fe catalyst demonstrates superior performance in the electrochemical co‐reduction of NO_x_ and R‐CHO, achieving oxime yields far exceeding those of other metal catalysts (Figure 4b). The yield of oxime (e.g., Ph‐CH═NOH) reaches ≈99%. Moreover, this electrochemical system exhibits broad substrate scope and excellent functional group tolerance, underscoring its great potential for practical applications in green chemical synthesis.

Metal–organic frameworks (MOFs), owing to their highly tunable structures, abundant metal active sites, and exceptional surface areas, have demonstrated unique advantages in electrocatalytic C–N coupling reactions in recent years.^[^ ^88^, ^89^ ^]^ MOFs can effectively adsorb and activate reactants such as NO_x_ and carbon‐containing species, while their adjustable pore structures facilitate the mass transport and enrichment of reaction intermediates, thereby enhancing the selectivity and efficiency of C–N bond formation. Li et al. uniformly dispersed Al‐containing MOF powder and polya‐crylonitrile (PAN) in a DMF solution as the precursor fluid to form NH_2_‐MIL‐53(Al)/PAN membranes. These membranes were then calcined at 800 °C under an Ar atmosphere, successfully synthesizing a high‐performance electrocatalyst with unsaturated coordination sites, named Al‐NFM (Figure 4c).^[^ ^90^ ^]^ This catalyst can in situ generate hydroxylamine (NH_2_OH) during the electrocatalytic reduction of NO and exhibit excellent catalytic activity for the synthesis of high‐value oximes. In an aqueous solution system, 2‐pyridinealdoxime was successfully synthesized with a FE of 49.8% and a yield of 92.1% (Figure 4d). Additionally, the durability of the catalyst was evaluated, and after 21 cycles, the catalyst's performance and structure remained stable, indicating that Al‐NFM has excellent long‐term stability (Figure 4e). Recently, the same group pioneered an interfacial coordination strategy based on MOF electrocatalysts.^[^ ^91^ ^]^ By constructing Zn–O bridges between graphite felt (GF) and a zeolitic imidazolate framework (ZIF‐7/CGF) (Figure 4f), they significantly enhanced the catalytic performance. In the electrosynthesis of 1‐methyl‐4‐piperidone oxime (1‐PDO), the engineered catalyst delivers outstanding results, achieving a FE of up to 75.9% and a yield of 73.1% (Figure 4g), dramatically outperforming the catalyst without Zn–O bridges, which exhibits only 10.7% FE and a 10.3% yield. Detailed mechanistic studies reveal that the introduction of Zn–O bridges not only promotes electron transfer but also induces the transformation of Zn sites into a distorted tetrahedral Zn–N_3_O coordination structure, thereby enhancing the adsorption and conversion of reaction intermediates. Furthermore, the ZIF‐7/CGF catalyst demonstrates excellent durability, maintaining high FE and product yield even after 16 continuous reaction cycles (Figure 4h).

Unlike MOF materials, which may suffer from metal cluster aggregation or uneven distribution of active sites, discrete cage structures are typically characterized by highly ordered, atomically dispersed metal active centers. This unique structural feature ensures that each metal site is located in an independent and well‐defined coordination environment, allowing for precise control over the catalytic reaction pathway. Thoi et al. successfully utilized a molecular copper boron‐imidazolate cage material, BIF‐29(Cu), to couple the electrochemical reduction of CO_2_RR with NO_3_RR for urea synthesis (Figure 4i),^[^ ^92^ ^]^ achieving exceptional catalytic performance with a selectivity of 68.5% and a current density of 424 µA cm^−2^. Through a combination of electrochemical analysis, in situ spectroscopy, and atomic‐scale simulations, the authors further proposed the reaction mechanism, namely CO_2_RR and NO_3_RR occur synergistically at distinct copper active sites, with the most favorable C–N coupling pathway driven by the condensation of *CO and NH_2_OH, ultimately resulting in urea formation (Figure 4j).

### 3.3 Multimetallic Catalysts

**Figure 5:**
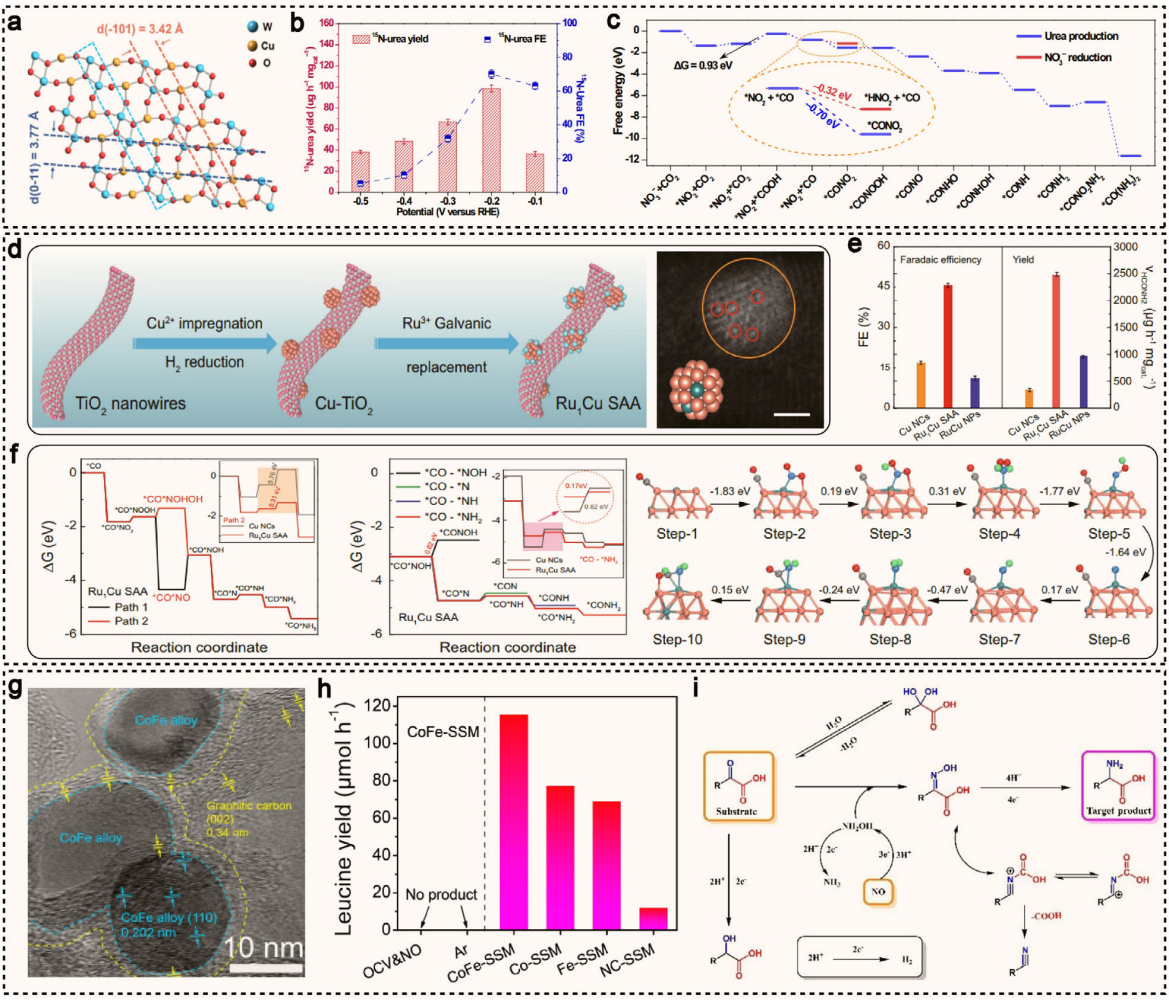
a) Atomic structure of CuWO_4_ (111) facet. b) ^15^N‐urea yield rates and FEs via integrated peak area from NMR data. c) Free‐energy diagram for urea production and NO_3_^−^ reduction on the CuWO_4_ (111) facet. Reproduced with permission.^[^ ^95^ ^]^ Copyright 2023, Springer Nature. d) Schematic illustration of preparation processes for Ru_1_Cu SAA catalyst, with Ti, O, Ru, and Cu atoms shown as pink, gray, blue, and orange, respectively. e) Highest formamide FEs and Yield of Cu NCs, Ru_1_Cu SAA, and RuCu NPs. f) DFT calculations of Ru_1_Cu SAA for the electrosynthesis of formamide. Reproduced with permission.^[^ ^96^ ^]^ Copyright 2023, Springer Nature. g) SEM of CoFe‐SSM. h) The yield of leucine under different test conditions with CoFe‐SSM and control specimens. i) Various pathways in the complex systems of electrosynthesis amino acids. Reproduced with permission.^[^ ^45^ ^]^ Copyright 2023, Wiley‐VCH.

Compared with monometallic catalysts, multimetallic catalysts can more effectively regulate the adsorption, activation, and transformation pathways of reaction intermediates through the synergistic interactions between different metal centers.^[^ ^93^ ^]^ For instance, one metal species may preferentially adsorb and activate nitrogen‐containing compounds, while another is more favorable for the activation of carbon‐based intermediates, thereby enabling spatially resolved and efficient coupling of C and N precursors. In addition, multimetallic catalysts can modulate the surface electronic structures and reaction energy barriers, enhancing both reaction selectivity and FE. This precise control over complex C–N bond formation pathways highlights the great potential of multimetallic catalysts in electrocatalytic C–N coupling reactions.^[^ ^94^ ^]^ Li et al. successfully synthesized a CuWO_4_ electrocatalyst with intrinsic bimetallic active sites via a hydrothermal method using tungstate and copper salts as the precursors (**Figure** **5**a).^[^ ^95^ ^]^ This catalyst demonstrates outstanding performance in the electrochemical co‐reduction of CO_2_ and NO_3_^−^, achieving a high urea yield of 98.5 ± 3.2 µg h^−1^ mg^−1^_cat_ and a FE of 70.1 ± 2.4% at −0.2 V versus RHE (Figure 5b). Mechanistic investigations reveal that the surface of CuWO_4_ facilitates early‐stage C–N coupling through the involvement of *NO_2_ and *CO intermediates. Owing to the alternately distributed W and Cu sites, which offer favorable adsorption properties and relatively positive formation potentials for these intermediates, CuWO_4_ effectively promotes their synergistic activation and coupling. This cooperative interaction not only increases the likelihood of C–N bond formation but also loweres the reaction energy barrier (Figure 5c). The synergism between *NO_2_ and *CO intermediates enhances selectivity and reduced overpotential, contributing to the catalyst's excellent electrocatalytic performance. This study offers new insights into catalyst design strategies for achieving efficient C–N coupling reactions.

Alloy catalysts have attracted widespread attention in C–N coupling reactions in recent years due to their combination of the high atomic utilization of SACs and the synergistic effects of bimetallic systems, offering unique catalytic advantages. For instance, Tan et al. prepared TiO_2_ nanowires with a diameter of ca. 10 nm as the catalyst support using a chemical dealloying method. Cu nanoclusters (Cu NCs) were then loaded onto the dealloyed TiO_2_ nanowires using a thermal reduction method.^[^ ^96^ ^]^ Subsequently, Ru single atoms were introduced onto the Cu NCs surface via galvanic replacement reactions, successfully obtaining the Ru_1_Cu single‐atom alloy catalyst (Ru_1_Cu SAA) (Figure 5d). In this catalytic system, using carbon monoxide and nitrite as raw materials, high FE of 45.65 ± 0.76% was achieved at −0.5 V (vs reversible hydrogen electrode) (Figure 5e), selectively synthesizing high‐value formamide. Mechanistic studies reveal that the adjacent Ru and Cu sites spontaneously couple *CO and *NH_2_ intermediates (Figure 5f), promoting the critical C–N coupling reaction, which endows the catalyst with excellent electrosynthesis performance for formamide. In another study, Li et al. prepared a CoFe‐PBA/PAN membrane by electrospinning a homogeneous solution of polyacrylonitrile (PAN) polymer and CoFe Prussian blue analog (CoFe‐PBA) under a high‐voltage electric field.^[^ ^45^ ^]^ The resulting membrane was then carbonized at 800 °C to obtain the CoFe‐SSM catalyst (Figure 5g). This catalyst was employed for the electrosynthesis of a series of α‐amino acids from nitric oxide (NO). In this process, leucine was produced with a yield of 115.4 µmol·h^−1^ and a FE of 32.4% (Figure 5h). The catalytic mechanism involves the rapid conversion of NO into NH_2_OH, which subsequently attacks α‐keto acids and undergoes hydrogenation to form amino acids (Figure 5i).

### 3.4 Metal‐Free Catalysts

**Figure 6:**
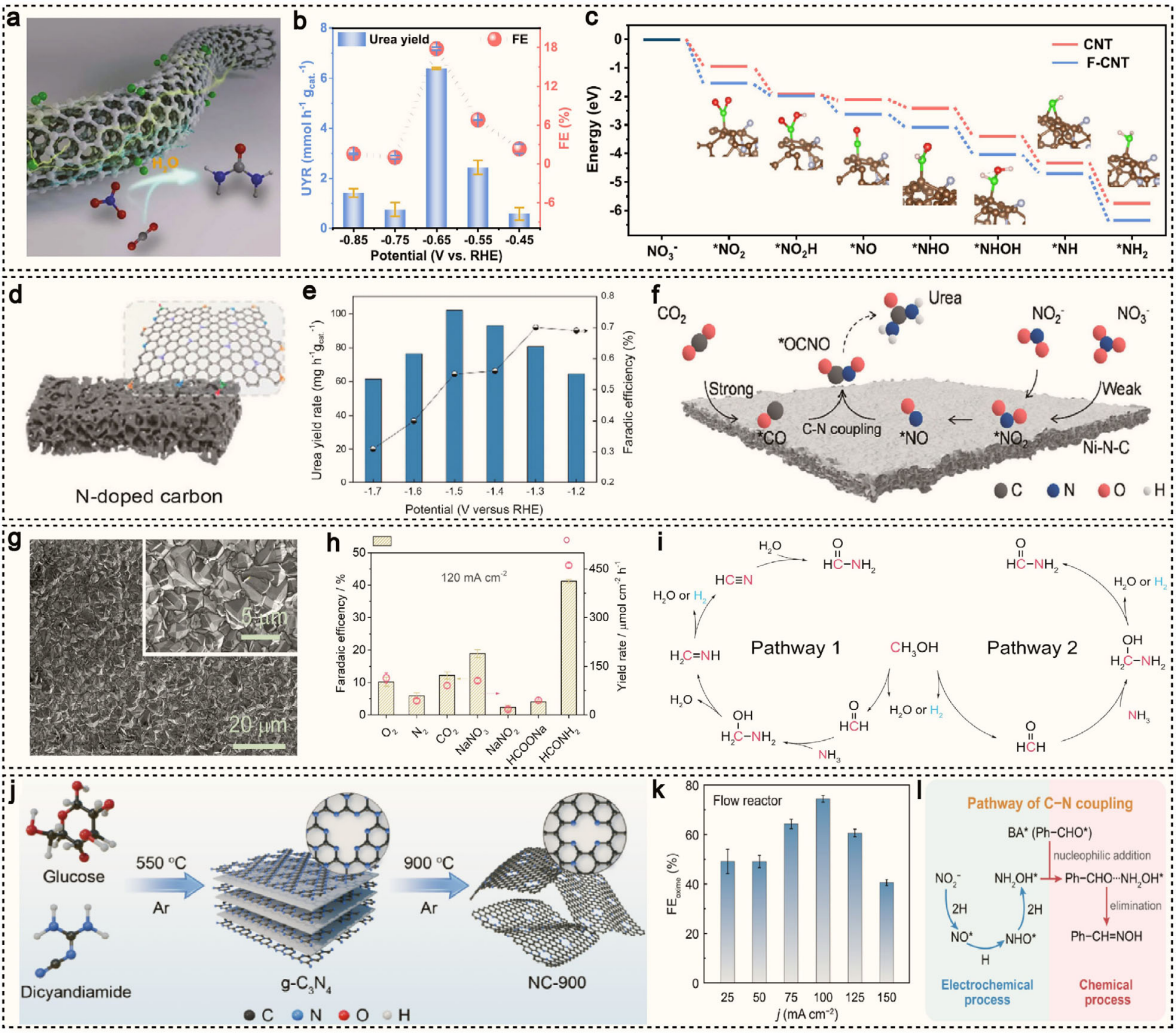
a) Schematic diagram of the structure of F‐CNT and the urea synthesis on the C‐MFC. b) Urea yield rates and FE values at different potentials for F‐CNT‐300 in CO_2_ saturated 0.1 M KNO_3_ electrolyte with CO_2_ flow. c) Potential energy diagrams of NO_3_^−^ reduction to *NH_2_. Reproduced with permission.^[^ ^49^ ^]^ Copyright 2022, Elsevier. d) Graphical illustration of the synthesis of porous N‐doped carbon. e) Urea yield rates and corresponding FEs of N‐C‐1000 at various potentials. f) Schematic diagram of the reaction pathway of electrocatalytic urea synthesis on Ni‐N‐C. Reproduced with permission.^[^ ^50^ ^]^ Copyright 2023, Wiley‐VCH. g) SEM images of BDD. h) FE and yield rate of oxidation products under the optimized conditions. i) Possible reaction pathways for the coupling of CH_3_OH and NH_3_ to HCONH_2_ via electrooxidation over BDD. Reproduced with permission.^[^ ^51^ ^]^ Copyright 2022, Wiley‐VCH. j) Schematic of the g‐C_3_N_4_‐assisted synthesis of NC‐T. k) FE_oxime_ over NC‐900 in 0.5 M K_2_CO_3_ with 10 mM BA and 0.3 M KNO_2_ in flow reactor. l) Schematic C−N coupling pathway for benzaldoxime electrosynthesis. Reproduced with permission.^[^ ^99^ ^]^ Copyright 2025, American Chemical Society.

Metal‐free catalysts have made significant strides in the realm of electrocatalytic C–N coupling reactions, emerging as environmentally friendly and highly efficient alternatives to conventional metal‐based systems.^[^ ^97^, ^98^ ^]^ Through the incorporation of heteroatoms such as nitrogen (N), boron (B), phosphorus (P), and fluorine (F) into carbon‐based frameworks, the electronic structures of these materials can be precisely tuned, enhancing their ability to adsorb and activate key intermediates, thus promoting C–N bond formation. For instance, fluorine‐doped carbon materials, due to their high electronegativity, can modulate interfacial charge distribution and stabilize reactive intermediates, exhibiting exceptional catalytic performance in the synthesis of urea, amides, and other nitrogen‐containing compounds. Similarly, nitrogen‐doped carbon materials leverage lone pair electrons to effectively facilitate nucleophilic or electrophilic coupling between carbon and nitrogen sources. Hu et al. introduced fluorine‐coated carbon nanotubes (F‐CNTs) as an efficient metal‐free electrocatalyst for the simultaneous activation of CO_2_ and NO_3_^−^ under ambient conditions, enabling the electrocatalytic synthesis of urea (**Figure** **6**a).^[^ ^49^ ^]^ This system demonstrates excellent performance, achieving a urea production rate of up to 6.36 mmol h^−1^ g_cat_^−1^ and a FE of 18.0% at −0.65 V versus (Figure 6b). DFT calculations further reveal the reaction pathways and energetics for the reduction of NO_3_^−^ to *NH_2_ on both undoped and fluorine‐doped carbon sites (Figure 6c). The results show that F doping significantly loweres the formation energy of key intermediates compared to pristine CNTs. In particular, the energy barrier for the transformation from *NO_2_ to *NH_2_ is consistently lower on F‐CNTs, indicating that the formation of *NH_2_ intermediates is more favorable on the fluorine‐doped surface, thereby promoting efficient urea synthesis. Chen et al. controllably synthesized a porous nitrogen‐doped carbon electrocatalyst with different nitrogen species ratios via the pyrolysis of coordination polymers and applied it to urea synthesis (Figure 6d).^[^ ^50^ ^]^ At a potential of −1.50 V, the catalyst achieves an excellent urea production rate of up to 610.6 mg h^−1^ g_cat_^−1^ (Figure 6e). Mechanistic studies indicate that the urea synthesis pathway originates from the coupling reaction of *NO and *CO, leading to the formation of the key intermediate *OCNO (Figure 6f).

Many noble metal materials (such as Pt, Pd, and Ru) are prone to oxidation and stripping at the anode under high current densities. To address this challenge, Zhang et al. employed a commercial boron‐doped diamond (BDD) electrode as the catalyst for the electrooxidation of methanol and NH_3_.^[^ ^51^ ^]^ The BDD film, densely coated on a silicon substrate (Figure 6g), served as an effective catalytic surface. In this system, formamide was synthesized via a one‐pot electrooxidation reaction using methanol and NH_3_. At a current density of 120 mA cm^−2^, the process achieves a formamide selectivity of 73.2% and a FE of 41.2% (Figure 6h), along with excellent reaction stability. Further mechanistic studies reveal that the formation of formamide may proceed via two potential pathways (Figure 6i). Methanol is oxidized to an aldehyde intermediate, which reacts with NH_3_ to form a hemiaminal intermediate. This intermediate is then converted to an imine, which undergoes further electrooxidation to a nitrile. The nitrile is subsequently hydrolyzed to yield formamide (Pathway 1). Methanol undergoes dehydrogenation or dehydration to directly produce formaldehyde, which reacts with NH_3_ to form a hemiaminal. This intermediate then undergoes further dehydrogenation or dehydration to produce formamide (Pathway 2). These two pathways collectively illustrate the possible mechanisms for C–N bond formation and formamide synthesis under electrocatalytic conditions. Recently, Hu et al. proposed a C−N coupling strategy based on multiple secondary bonding interactions, achieving efficient synthesis of benzaldoxime using a nitrogen‐doped graphene‐like carbon catalyst (NC).^[^ ^99^ ^]^ They used glucose and dicyandiamide as carbon and nitrogen sources, respectively, and obtained g‐C_3_N_4_ nanosheets via an in situ polymerization–pyrolysis process, serving as a robust sacrificial template. Subsequently, high‐temperature decomposition yielded the graphene‐like NC‐T material (Figure 6j). Notably, the NC catalyst achieved a high FE of 73 ± 1% and a benzaldoxime electrosynthesis yield rate of 6.8 ± 0.1 mol h^−1^ m^−2^ at an economically viable current density of 0.1 A cm^−2^ (Figure 6k). Mechanistic analysis revealed that the electrosynthesis pathway of benzaldoxime is a multi‐step cascade electrochemical–chemical process (Figure 6l): NO_2_^−^ first adsorbs on the NC‐900 surface and loses one electron to form NO_2_*, which is then sequentially reduced to NO*, NHO*, NH_2_O* and NH_2_OH*. Subsequently, NH_2_OH* undergoes a rapid and spontaneous nucleophilic addition–elimination reaction with surface‐enriched benzaldehyde (BA*), producing benzaldoxime.

## 4 Organonitrogen Compounds from Electrochemical C–N Coupling

### 4.1 Urea

**Figure 7:**
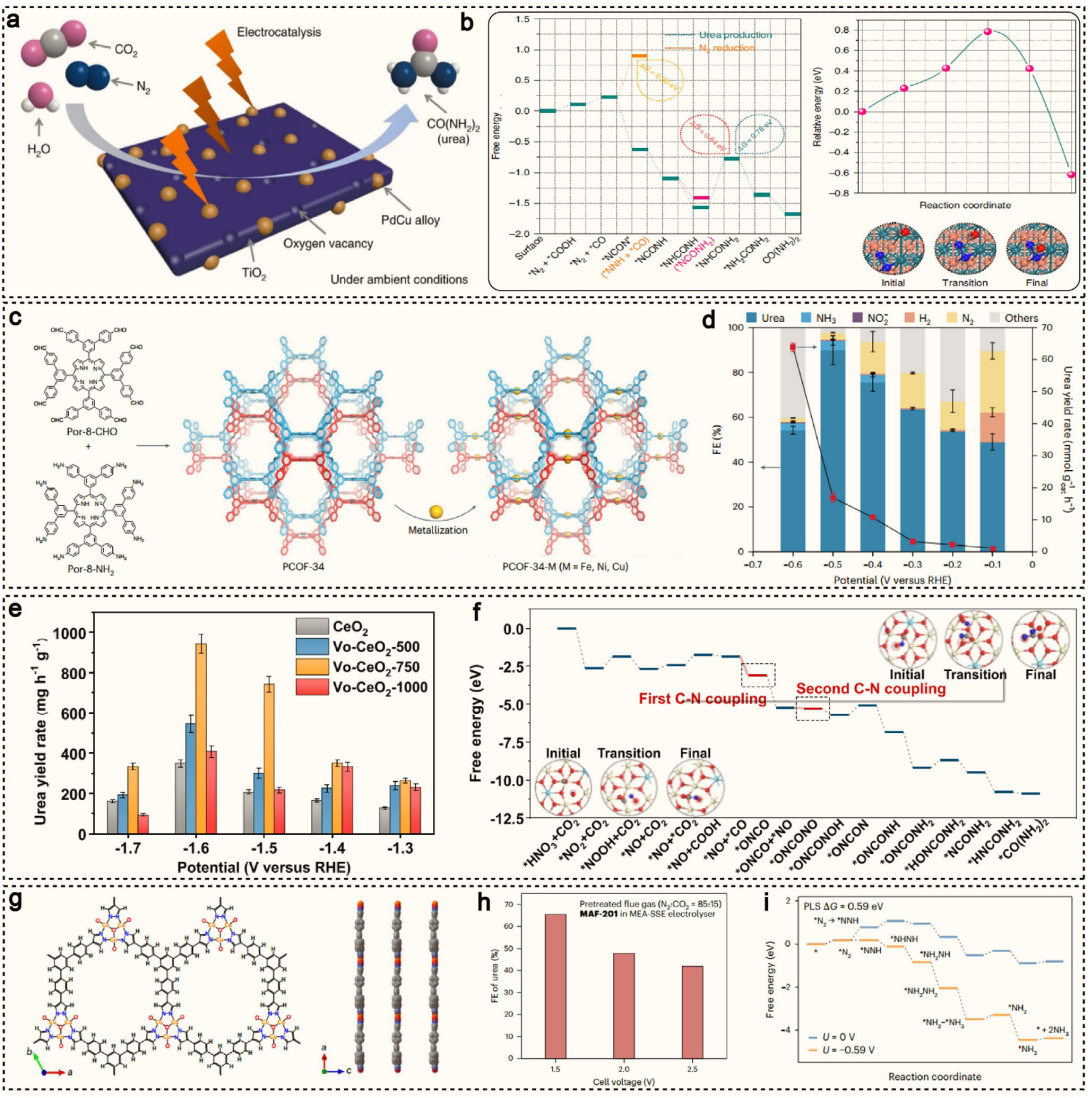
a) Scheme of the C−N coupling to urea by Pd_1_Cu_1_‐TiO_2_. b) Free energy diagram of urea production and the reaction pathway of *NCON* formation. The structures of the initial, transition and final states along with the *NCON* formation, are also presented. Reproduced with permission.^[^ ^63^ ^]^ Copyright 2020, Springer Nature. c) Synthesis and structural representation of PCOF‐34 and subsequent synthesis of PCOF‐34‐M (M = Fe, Ni, Cu) through metallization. d) Potential‐dependent urea production rate and FE for PCOF‐34‐Fe. Reproduced with permission.^[^ ^108^ ^]^ Copyright 2025, Springer Nature. e) Urea yield rates of CeO_2_, Vo‐CeO_2_‐500, Vo‐CeO_2_‐750, and Vo‐CeO_2_‐1000 at various applied potentials. f) Free energy diagram of urea production on Vo‐enriched CeO_2_. Reproduced with permission.^[^ ^109^ ^]^ Copyright 2022, American Chemical Society. g) Top view of the layer showing the {Cu_3_(*µ*_3_‐O)} nodes and side view of the layers in MAF‐201. h) FE_urea_ values of MAF‐201 at different cell voltages in the MEA‐SSE electrolyzer by using pretreated flue gas as feedstock. i) Free energy diagram of NH_3_ generation on MAF‐201 under flow‐cell conditions (potential limiting step). Reproduced with permission.^[^ ^110^ ^]^ Copyright 2025, Springer Nature.

Traditional urea synthesis relies on the Haber–Bosch and Bosch–Meiser process, which operates at high temperatures (150–200 °C) and pressures (150–250 bar) due to the high bond energy of the N≡N triple bond (940.95 kJ mol^−1^).^[^ ^100^, ^101^ ^]^ This method is energy‐intensive, and places considerable strain on equipment and the environment, and generates large amounts of CO_2_, contributing significantly to environmental pollution. As a result, traditional methods face major challenges in terms of high energy consumption and environmental impact. In contrast, the electrocatalytic co‐reduction of CO_2_ and nitrogen sources (such as NO_3_^−^ or N_2_) offers a greener alternative for urea synthesis.^[^ ^102^, ^103^, ^104^, ^105^, ^106^, ^107^ ^]^ This approach enables urea production under mild conditions, avoiding the high energy demands and environmental burdens associated with traditional methods. For instance, Wang et al. developed an electrocatalytic method for directly coupling N_2_ and CO_2_ in aqueous solution to synthesize urea under ambient conditions, using PdCu as the catalyst (**Figure** **7**a).^[^ ^63^ ^]^ At −0.4 V versus RHE, the FE was found to be 8.92%. DFT calculations indicated that the chemical reaction between *N═N and CO occurs spontaneously, forming a C–N bond (Figure 7b). The presence of *N═N enhances CO_2_ reduction, and the reduced CO can subsequently react with *N═N to form urea, showcasing high catalytic activity and selectivity. Compared to the N≡N bond, the N═O bond offers distinct advantages in electrocatalytic urea synthesis. The N═O bond has a lower bond energy (204 kJ mol^−1^), making it easier to break and lowering the reaction energy barrier. This facilitates the formation of more reactive intermediates, enhancing the efficiency of C−N coupling. Therefore, utilizing the N═O bond in NO_3_^−^ can reduce energy consumption and improve reaction selectivity and yield. Cui et al. designed a porous, 3D interpenetrated covalent organic framework (COF) based on metal porphyrins and used it for the electrocatalytic production of urea (Figure 7c), utilizing NO_3_^−^ and CO_2_ as feedstocks.^[^ ^108^ ^]^ PCOF‐34‐Fe, which incorporates dual iron sites, demonstrates outstanding electrocatalytic performance, achieving a FE of 90.0%, a urea yield of 135.6 mmol g^−1^_cat_·h^−1^, and nitrogen and carbon selectivity of 92.4% and 100%, respectively (Figure 7d). Both experimental data and theoretical calculations reveal that the Fe‐N_4_ sites in the porphyrin structure effectively facilitate the conversion of CO_2_ to *CO and NO_3_^−^ to *NH_2_. Furthermore, the spatial arrangement of the twin iron sites overcomes the disordered transfer of intermediates, enabling efficient C−N coupling.

In the urea synthesis process, the hydrogenation of intermediate products hinders the efficient formation of urea. To address this issue, Wang et al. designed an oxygen vacancy‐rich CeO_2_ electrocatalyst, which stabilizes the key intermediate *NO by inserting vacancies, thereby promoting the subsequent C−N coupling process rather than protonation.^[^ ^109^ ^]^ The introduction of oxygen vacancies customizes the catalyst support into an effective electrocatalyst, achieving a urea yield of up to 943.6 mg h^−1^ g^−1^ (Figure 7e), outperforming some noble metal‐based electrocatalysts. Mechanistic studies reveal that the hydrogenation of *NO_2_ is the rate‐determining step (0.77 eV), which is nearly identical to the hydrogenation of *CO_2_ (0.68 eV). The formation of the second C−N bond arises from the coupling of *OCNO with *NO species, with a low energy barrier of 0.24 eV (Figure 7f). Subsequently, the hydrogenation reaction progresses, leading to urea formation. Recently, Liao et al. designed a catalyst composed of nanosheets with a 2D metal‐azole framework, featuring a cyclic heterotrimetal cluster, MAF‐201 (Figure 7g), which were used to produce urea from pretreated flue gas.^[^ ^110^ ^]^ The FE of urea reached 65.5% (Figure 7h), with no liquid by‐products such as NH_3_ formed. Studies show that the proton‐limited environment established in an electrolyser equipped with a porous solid‐state electrolyte effectively suppresses hydrogen evolution and the excessive hydrogenation of N_2_ to NH_3_ (Figure 7i). This environment, in turn, favors the C−N coupling reaction between *CO_2_ and *NHNH.

### 4.2 Oximes

**Figure 8:**
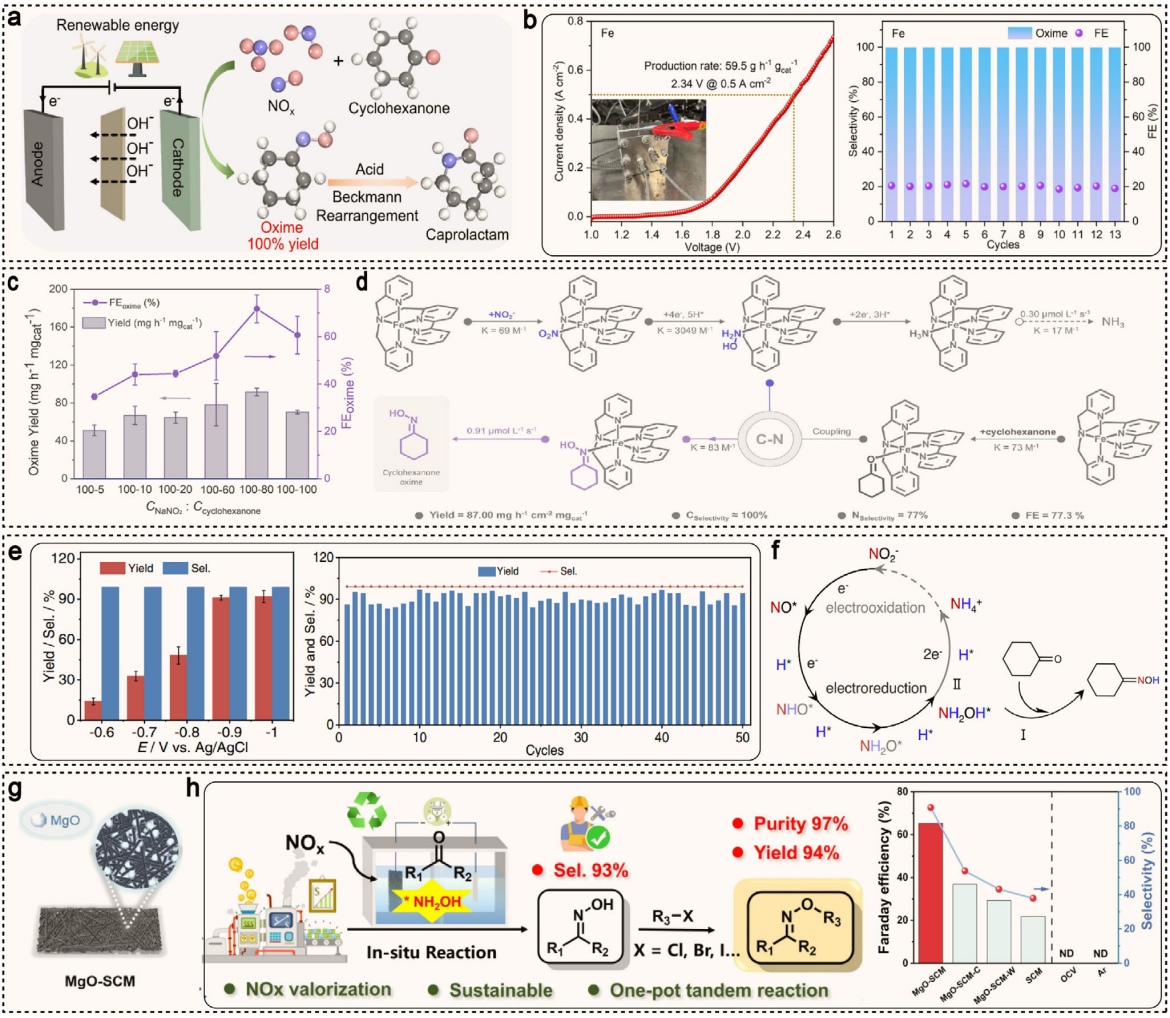
a) Schematic illustration of the sustainable caprolactam production involving the direct electrocatalytic synthesis of cyclohexanone oxime from NO_x_ and CYC. b) The LSV curves in an alkaline flow cell and stability measurement of Fe electrocatalyst for 13 cycles. Reproduced with permission.^[^ ^114^ ^]^ Copyright 2023, Wiley‐VCH. c) Effects of substrate concentration (mmol L^−1^) ratio on cyclohexanone oxime yield and FE. d) Proposed mechanism for cyclohexanone oxime and ammonia electrosynthesis by FeBPAbipyH. Reproduced with permission.^[^ ^115^ ^]^ Copyright 2025, Wiley‐VCH. e) Potential‐dependent cyclohexanone oxime yield and selectivity and time‐dependent cyclohexanone conversion and cyclohexanone oxime yield. f) Schematic illustration of the cyclohexanone oxime generation pathway. Reproduced with permission.^[^ ^116^ ^]^ Copyright 2023, Springer Nature. g) Graphical illustration of synthetic MgO‐SCM. h) Scheme of reaction routes of oxime ethers production and FE and selectivity of 4‐cyanobenzaldoxime over different catalysts under various conditions. Reproduced with permission.^[^ ^117^ ^]^ Copyright 2024, Wiley‐VCH.

The current synthesis of oximes mainly involves the reaction of aldehydes or ketones with hydroxylamine.^[^ ^111^ ^]^ Traditional methods require heating and catalysts, often associated with high energy consumption, safety risks, and the use of toxic solvents, limiting their application in green chemistry. For instance, cyclohexanone oxime is a key precursor for the synthesis of caprolactam, used in the production of nylon‐6. The traditional synthesis method involves the reaction of cyclohexanone (CYC)‐hydroxylamine and cyclohexanone ammoxidation methodologies. These methods require complex steps, high temperatures, precious metal catalysts, and the use of toxic SO_2_ or H_2_O_2_. In contrast, electrocatalytic C−N coupling for oxime synthesis offers significant advantages.^[^ ^112^, ^113^ ^]^ By electrocatalytically reducing NO_x_ or other nitrogen sources to generate NH_2_OH, which then reacts with ketones to form oxime intermediates, this method operates under mild conditions, with low energy consumption and is environmentally friendly. Zou et al. discovered that iron can serve as an ideal electrocatalyst for the efficient synthesis of cyclohexanone oxime from NO_x_ and CYC via electrocatalysis, achieving a near 100% yield without directly using the explosive NH_2_OH (**Figure** **8**a).^[^ ^114^ ^]^ In a flow cell, at a current density of 500 mA cm^−2^ and a voltage of 2.34 V, the yield of cyclohexanone oxime reached 59.5 g h^−1^ g_cat_^−1^. Furthermore, in a 10 mL 0.5 M K_2_CO_3_ electrolyte, under conditions of 0.1 M CYC, 1 M KNO_3_, and 0.30 V versus RHE, the selectivity and FE of cyclohexanone oxime remained ≈100% and ≥20% over 13 cycles, demonstrating the excellent stability of the Fe electrocatalyst (Figure 8b). DFT calculations and in situ characterizations reveal that the ability of iron to adsorb NH_2_OH and cyclohexanone is the key factor responsible for the high efficiency of the reaction. Similarly, Wu et al. designed an iron‐based molecular catalyst, iron bis(pyridyl)amine‐bipyridine (FeBPAbipyH), with well‐defined active sites, and anchored it onto conductive multi‐walled carbon nanotubes (MWCNTs@CP) to explore its application in cyclohexanone oxime synthesis.^[^ ^115^ ^]^ Under optimal conditions (100 mM NO_2_^−^ and 80 mM cyclohexanone, potential at −0.40 V vs RHE), the yield of cyclohexanone oxime reached 87.00 mg h^−1^ cm^−2^ mg_cat_^−1^, with a FE of 77.3% (Figure 8c). Mechanistic studies reveal that the FeBPAbipyH framework exhibits strong interactions with *NH_2_OH and significant binding affinity with both cyclohexanone and NO_2_^−^. This facilitates the reduction of NO_2_^−^ to NH_2_OH rather than NH_3_, promoting the C−N coupling reaction (Figure 8d). Zhang et al. employed an inexpensive Cu‐S catalyst, avoiding complex reaction steps, precious metal catalysts, and the use of H_2_SO_4_/H_2_O_2_.^[^ ^116^ ^]^ At a potential of −0.9 V (vs Ag/AgCl), the yield of cyclohexanone oxime reached 92%, with a selectivity of 99% (Figure 8e). After 50 cycles, the catalyst maintained its high performance, demonstrating its durability. Mechanistic studies reveal that the reaction proceeds through the reduction of NO_2_^−^ to form NH_2_OH*, followed by condensation of NH_2_OH* with cyclohexanone to generate cyclohexanone oxime (Figure 8f). Moreover, this method is also applicable to the synthesis of other oximes, showcasing its broad applicability. Recently, Li et al. developed a novel electrocatalyst using Mg‐MOF/polymer self‐standing carbon‐nanofibers membrane (MgO‐SCM) as the support (Figure 8g), for one‐pot tandem electrochemical synthesis of oxime ether.^[^ ^117^ ^]^ In this system, NO is first converted to NH_2_OH, which then spontaneously attacks aldehydes to form oximes. The catalyst exhibits high selectivity (93%) and FE (65.1%) for the synthesis of 4‐cyanobenzaldoxime (Figure 8h). This oxime further reacts with benzyl bromide, and the oxime ether is directly obtained with 97% purity via a convenient filtration separation.

### 4.3 Amino Acids

**Figure 9:**
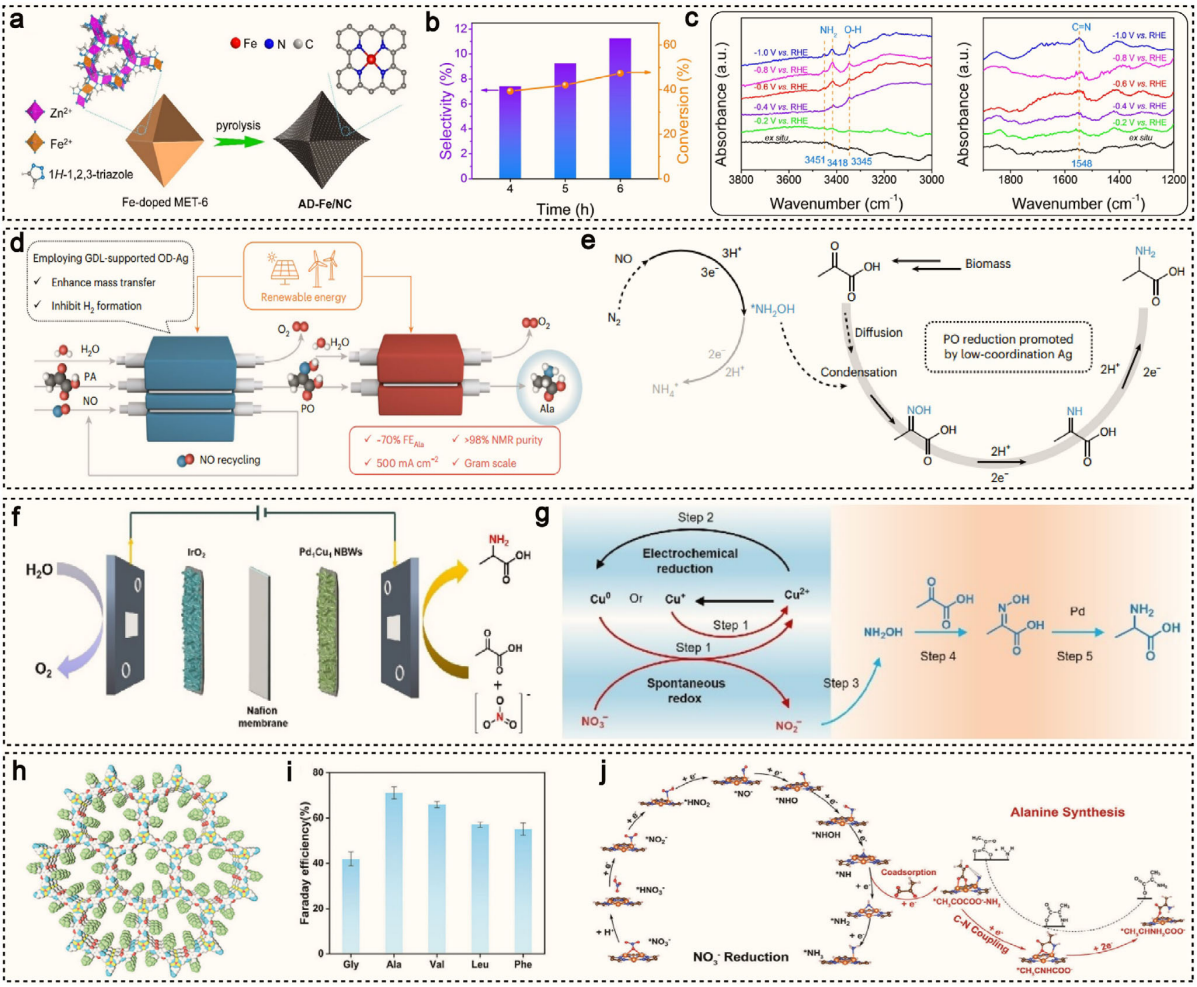
a) Schematic diagram showing the preparation of AD‐Fe/NC. b) The selectivity of valine and the conversion of 3‐methyl‐2‐oxobutanoic acid during the electrosynthesizing valine process. c) In situ SRIR results in the range of 3000−3800 cm^−1^ and 1200−1900 cm^−1^ versus potentials for AD‐Fe/NC under working conditions. Reproduced with permission.^[^ ^121^ ^]^ Copyright 2023, Wiley‐VCH. d) Proposed spatially decoupled, upgraded two‐pot route to enhance the space‐time yield of alanine. e) Schematic illustration of the alanine generation pathway on the OD‐Ag surface. Reproduced with permission.^[^ ^64^ ^]^ Copyright 2023, Springer Nature. f) Schematic diagram illustrating the electrochemical coupling of biomass‐derived pyruvic acid and NO_3_^−^ to produce alanine. g) The reaction mechanism for the coupling reaction. Reproduced with permission.^[^ ^122^ ^]^ Copyright 2023, Wiley‐VCH. h) The graphical illustration depicts the synthesis of F‐Cu_3_‐OF. i) The optimized FEs of glycine, alanine, valine, leucine, and phenylalanine for F‐Cu_3_‐OF. j) The reaction pathway for the generation of alanine form NO_3_^−^ and CH_3_COCOO^−^ on the trinuclear Cu(I) group (Cu^I^_3_) of reduced F‐Cu_3_‐OF. Reproduced with permission.^[^ ^123^ ^]^ Copyright 2024, Wiley‐VCH.

Commonly used methods for amino acid synthesis include enzymatic reactions, microbial fermentation, and chemical synthesis.^[^ ^118^, ^119^, ^120^ ^]^ While these methods can efficiently synthesize amino acids, they often rely on petroleum‐based raw materials, long reaction cycles, and significant energy consumption. Additionally, they generate waste, have high costs, and are environmentally unfriendly. Traditional chemical synthesis methods, such as the Strecker reaction, require the use of toxic cyanides and ammonia, and involve high energy consumption, leading to environmental pollution. In contrast, electrocatalytic C−N coupling for amino acid synthesis occurs under mild conditions by reducing CO_2_ or NO_x_ and reacting with keto acids, offering lower energy consumption and higher selectivity. This method does not rely on petroleum‐based raw materials and uses renewable resources, reducing environmental impact and the use of toxic chemicals, making it a greener and more sustainable alternative. For instance, Li et al. achieved the synthesis of α‐amino acids for the first time in a keto acid hydrogenation coupled NO_x_ reduction system, using atomically dispersed Fe supported on nitrogen‐doped carbon (AD‐Fe/NC) as the catalyst (**Figure** **9**a).^[^ ^121^ ^]^ At −0.6 V versus RHE, the yield of valine is 32.1 µmol mg_cat_^−1^, with a corresponding selectivity of 11.3% (Figure 9b). In situ synchrotron radiation infrared spectroscopy (SRIR) was used to identify the key intermediate C═N–OH (Figure 9c), and both the experimental and spectral results confirmed that the critical step in amino acid production is the formation of NH_2_OH during NO_x_ reduction. NH_2_OH then rapidly attacks the α‐carbon of the keto acid, forming the key intermediate oxime, which undergoes subsequent hydrogenation to produce the amino acid.

However, due to the complexity of the reaction pathways, the FE of the system reported by Li is relatively low. Zhang et al. designed an oxide‐derived silver catalyst that can electrochemically synthesizes alanine at room temperature using NO and pyruvic acid (PA) as feedstocks (Figure 9d).^[^ ^64^ ^]^ The total FE for the synthesis of alanine from NO and PA reached 70%, with a purity exceeding 98%. Mechanistic studies reveal that, on the OD‐Ag electrode surface, NO is reduced to the *NH_2_OH intermediate, which then spontaneously condenses with PA to form the oxime intermediate (Figure 9e). Under the promotion of low‐coordination silver, the pyruvate oxime (PO) is reduced to alanine via the imine pathway. Wang et al. designed and synthesized a PdCu nano‐bead‐wires (PdCu NBWs) electrocatalyst, which successfully electrochemically coupled biomass‐derived pyruvic acid (PA) and NO_3_^−^ to produce alanine (Figure 9f).^[^ ^122^ ^]^ Mechanistic studies reveal that adsorbed NO_3_^−^ undergoes a spontaneous redox reaction with Cu, forming Cu^2+^ and NO_2_^−^. The NO_2_^−^ is then hydrogenated to form NH_2_OH, while Cu^2+^ is reduced to Cu^+^, enabling a new hydrogenation cycle (Figure 9g). Subsequently, the generated NH_2_OH spontaneously condenses with PA to form an intermediate, effectively suppressing ammonia formation. Finally, through the Pd sites, a 4‐electron and 4‐proton transfer process reduces the resulting oxime electrochemically to the desired alanine product. Recently, Jiang et al. synthesized an COF containing a trinuclear copper cluster (F‐Cu_3_‐OF) (Figure 9h).^[^ ^123^ ^]^ By introducing a large number of hydrophobic perfluoroalkyl groups into its pores, they successfully inhibited the competitive HER by preventing the large, polar H^+^ cation from leaving the F‐Cu_3_‐OF framework. This modification also enhances the adsorption of smaller, less polar substrates like NO_3_^−^ and keto acids at the active sites. The shorter distance between adjacent copper atoms in the F‐Cu_3_‐OF allows simultaneous activation of NO_3_^−^ and keto acids, promoting subsequent synergistic and efficient C−N coupling. As a result, it enables the efficient electrochemical production of various amino acids, including glycine, alanine, leucine, valine, and phenylalanine, with FE ranging from 42% to 71% and yields from 187 to 957 µmol cm^−2^ h^−1^ (Figure 9i). DFT calculations indicate that the hydrogen bond interaction between *CH_3_COCOO^−^ and *NH_2_ further induces a thermodynamically favorable C−N coupling reaction, leading to the preferential formation of *CH_3_CNHCOO^−^ (Figure 9j).

### 4.4 Amides

**Figure 10:**
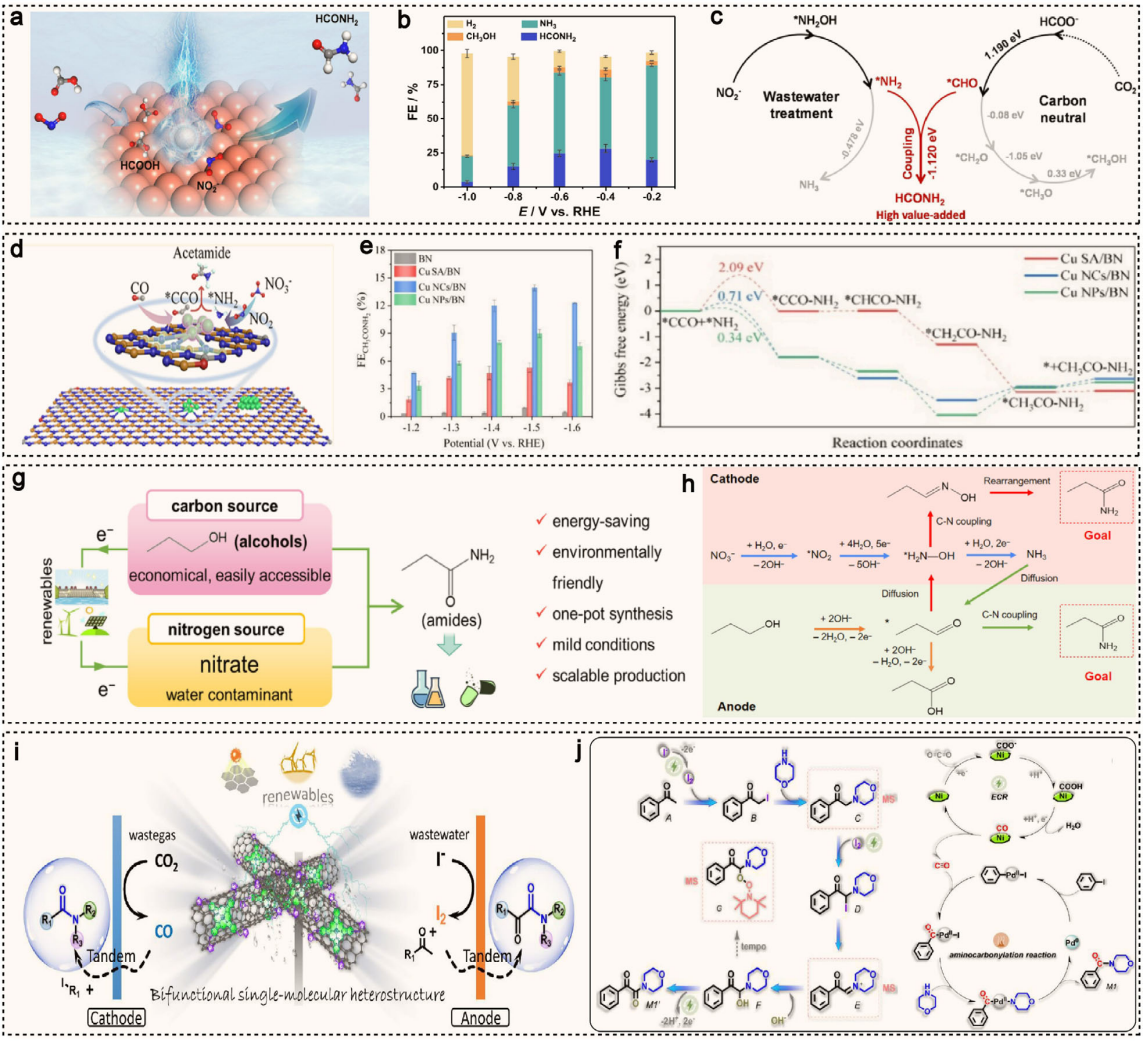
a) The graphical illustration depicting the synthesis formamide using copper as a catalyst. b) Potential‐dependent FE distribution of electrolysis products. c) Schematic illustration of formamide generation pathway on the Cu nanocubes surface. Reproduced with permission.^[^ ^129^ ^]^ Copyright 2022, American Chemical Society. d) The graphical illustration of the acetamide synthesis using Cu/BN catalysts. e) FE of acetamide. f) Free energy diagrams of C−N coupling to CH_3_CONH_2_ on Cu SA/BN, Cu NCs/BN, and Cu NPs/BN. Reproduced with permission.^[^ ^130^ ^]^ Copyright 2024, American Chemical Society. g) Schematic diagram of the electrochemical cathode‐anode coupling strategy to produce propanamide using NO_3_^−^ and n‐propanol. h) Proposed reaction pathway of cathode‐anode coupling to produce propenamide. Reproduced with permission.^[^ ^131^ ^]^ Copyright 2025, American Chemical Society. i) Schematic diagram of the electrolysis‐paired tandem synthesis system with integrated dual modules for the concurrent syntheses of amides and α‐ketoamides, by utilizing renewables to drive the tandem processes of electrocatalysis and chemocatalysis. j) Proposed mechanisms for the anodic and cathodic tandem. Reproduced with permission.^[^ ^132^ ^]^ Copyright 2025, Wiley‐VCH.

Amides are an important class of organic compounds, widely used in industries such as pharmaceuticals, agrochemicals, plastics, dyes, and emulsifiers, with significant roles in the production of antibiotics, anticancer drugs and polyamides.^[^ ^124^, ^125^, ^126^ ^]^ Traditional methods for amide synthesis often rely on high temperature and pressure conditions, and the use of expensive and toxic catalysts and solvents, resulting in high energy consumption and potential environmental pollution and safety risks.^[^ ^127^, ^128^ ^]^ Moreover, many traditional methods depend on organic acid derivatives, making the synthesis process complex and inefficient. With the development of green chemistry, electrocatalytic amide synthesis using renewable resources has become a research focus. This method enables the construction of C−N bonds under mild conditions using abundant and inexpensive carbon‐ and nitrogen‐containing feedstocks, such as formic acid and nitrates, reducing energy consumption and avoiding environmental pollution, thereby demonstrating significant potential for green chemistry. For instance, Zhang et al. reported an electrocatalytic method for the conversion of formic acid and nitrite into high‐value formamide using copper as a catalyst under ambient conditions (**Figure** **10**a).^[^ ^129^ ^]^ In aqueous media, an optimal FE_HCONH2_ of 29.7% was achieved at −0.4 V versus RHE (Figure 10b). A series of in situ and quasi in situ experiments, along with theoretical simulations, reveal that low‐coordination Cu sites can spontaneously couple *CHO and *NH_2_, forming the key C−N bond, which leads to high‐performance electrochemical synthesis of formamide (Figure 10c).

To meet the synthesis needs of more complex functional molecules, researchers have gradually expanded their focus to higher carbon number amides, such as acetamide and propionamide. Wu et al. prepared Cu/BN catalysts with varying Cu atomic content, using boron nitride as the support, for the co‐reduction of CO and NO_3_^−^ to synthesize acetamide (Figure 10d).^[^ ^130^ ^]^ At an industrial‐level current density of 178 mA cm^−2^, they achieved a record‐breaking acetamide production rate of 137.0 mmol h^−1^ g_cat_^−1^, with a maximum FE of 13.9% (Figure 10e). DFT calculations indicate that the hydrogenation of *CH_2_CONH_2_ to *CH_3_CONH_2_ on the Cu NCs/BN catalyst is thermodynamically more favorable, with a lower free energy change of 0.45 eV, compared to a free energy change of 0.55 eV on Cu NPs/BN (Figure 10f). The computational results highlight the synergistic effect between Cu nanoclusters and B sites in Cu NCs/BN, which significantly promotes the co‐reduction of CO and NO_3_^−^, leading to the successful synthesis of acetamide. Lu et al. designed a novel and sustainable cathode‐anode synergistic electrocatalytic system using Co_3_O_4_/SiC and Ti as the cathode and anode catalysts, respectively, for the conversion of NO_3_^−^ and n‐propanol (CH_3_CH_2_CH_2_OH) into propionamide under ambient conditions (Figure 10g).^[^ ^131^ ^]^ At a current density of 650 mA cm^−2^, the C−N bond electrosynthesis efficiency reached 986.13 µmol (cm^2^·h)^−1^. Mechanistic studies reveal that the key reaction intermediates are NH_2_OH generated at the cathode and propionaldehyde produced at the anode, which further react at the cathode to form propionamide (Figure 10h).

Recently, Zhu et al. developed a metalloenzyme‐inspired single‐molecular heterostructured catalyst (MimNiPc/CNT), constructed by hybridizing carbon nanotubes (CNTs) with methylimidazole‐functionalized nickel phthalocyanine (MimNiPc) via strong π‐π hetero‐stacking and charge‐transfer interactions.^[^ ^132^ ^]^ Building on this, they designed an innovative electrolysis‐paired tandem synthesis system for co‐production of amides and α‐ketoamides. In this system, the cathode carries out electrocatalytic CO_2_ reduction to generate CO in situ, which is subsequently employed in aminocarbonylation reactions for amide synthesis (Figure 10i). Concurrently, the anode enables the formation of α‐ketoamides through an iodine (I_2_)‐mediated keto‐amidation tandem reaction. Using detailed isotope labeling and mass spectrometry tracking, the research team comprehensively elucidated the reaction mechanism, which involves metal migration, CO insertion, nucleophilic substitution, and reductive elimination (Figure 10j). The tandem reactions at both electrodes were further revealed to proceed through a sequential and synergistic interplay between electrocatalysis and chemocatalysis.

### 4.5 Amines

**Figure 11:**
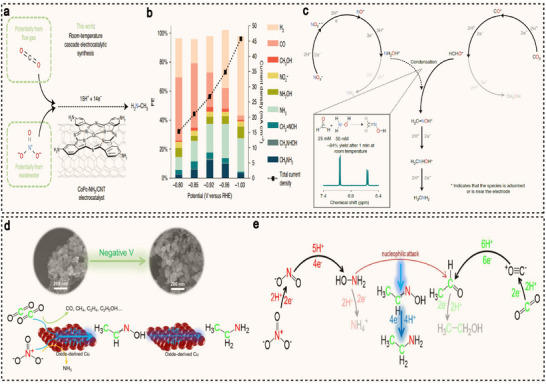
a) The graphical illustration depicting the one‐pot electrosynthesis of methylamine from inorganic wastes. b) Potential‐dependent product distribution (FE) and total current density. c) The proposed reaction pathway of the eight‐step cascade electrosynthesis of methylamine from CO_2_ and NO_3_^−^ catalyzed by CoPc‐NH_2_/CNT. Reproduced with permission.^[^ ^112^ ^]^ Copyright 2021, Springer Nature. d) The graphical illustration depicting the electrochemical co‐reduction of CO_2_ and NO_3_^−^ to synthesize acetaldoxime and ethylamine. e) Proposed reaction pathway to form acetaldoxime and ethylamine from electrochemical co‐reduction of CO_2_ and NO_3_^−^. Reproduced with permission.^[^ ^136^ ^]^ Copyright 2022, Elsevier.

Amines are widely used in various fields, including pharmaceuticals, agriculture, dyes, polymers, and chemical synthesis.^[^ ^133^, ^134^, ^135^ ^]^ However, traditional methods for synthesizing amines still face numerous challenges. These conventional methods often rely on complex catalytic reactions, requiring high temperatures and pressures, and the use of toxic and expensive catalysts or reducing agents, resulting in high energy consumption and low reaction efficiency. For instance, the industrial synthesis of methylamine typically involves high‐temperature and high‐pressure conditions, with methanol derived from fossil fuels and ammonia. In contrast, electrochemical reduction using CO_2_ and NO_3_^−^ as carbon and nitrogen sources may offer a more sustainable process for the green production of methylamine and other related organic nitrogen compounds. However, a major challenge in the synthesis process is designing an efficient C−N coupling step that effectively links the reduction pathways of both precursors, enabling the high‐efficiency synthesis of the target product. Wang et al. developed a cascade electrochemical reaction, catalyzed by a cobalt β‐tetraaminophthalocyanine molecular catalyst supported on carbon nanotubes (CoPc‐NH_2_/CNT), which successfully converts carbon dioxide and nitrate into methylamine for the first time (**Figure** **11**a).^[^ ^112^ ^]^ Under ambient conditions, methylamine is produced through a highly cooperative “one‐pot” process, where each product molecule undergoes a total transfer of 14 electrons and 15 protons, with an overall FE of 13% (Figure 11b). Mechanistic studies indicate that the reaction involves two key intermediates, i.e., NH_2_OH (from nitrate reduction) and formaldehyde (from CO_2_ reduction). These intermediates rapidly undergo effective C−N coupling to form a transitional formaldehyde oxime, which is then hydrogenated and dehydrated to yield methylamine (Figure 11c). The same research team further developed an amine‐aldehyde coupling reaction and successfully synthesized ethylamine under ambient conditions (Figure 11d).^[^ ^136^ ^]^ The reaction is catalyzed by oxide‐derived Cu nanoparticles and involves the coupling reduction of nitrate and CO_2_. However, due to the multi‐step nature of the reaction process, the FE for ethylamine is only 0.15%. Mechanistic studies suggest that in the overall reaction for CO_2_ and NO_3_^−^ to form ethylamine, CO_2_ and NO_3_^−^ are first reduced to generate acetaldehyde and NH_2_OH, respectively. These two intermediates then undergo a chemical reaction to form acetaldoxime, which is subsequently reduced to ethylamine (Figure 11e). Although some successful studies have demonstrated the possibility of electrocatalytic amine synthesis, the current technology still faces challenges such as low reaction efficiency and poor selectivity. In particular, the management of intermediates in multi‐step reactions and the precise control of the C−N coupling reactions remain technical bottlenecks. Future research should focus on developing new catalysts and optimizing reaction pathways to improve yields and enhance FE.

### 4.6 Others

In addition to the commonly known nitrogen‐containing organic compounds, electrocatalytic C−N coupling can also be used to synthesize other types of nitrogen‐containing organic compounds. For instance, recent studies have explored the electrocatalytic C−N coupling reaction between nitrate and alkynes or aromatic compounds to synthesize organic nitriles.^[^ ^137^, ^138^, ^139^ ^]^ This process typically involves the reduction of nitrate to generate NO_2_ or amino intermediates, which then undergo efficient C−N coupling with alkynes or aromatic compounds to form nitrile compounds. Nitriles are widely used in pharmaceuticals, pesticides, plastics, and dyes. Similarly, electrocatalytic C−N coupling reactions between nitrate and aromatic compounds have been employed to synthesize aniline and its derivatives,^[^ ^140^, ^141^, ^142^ ^]^ which would play an important role in dye, polymer, and pharmaceutical synthesis. By precisely controlling reaction conditions, efficient C−N bond formation and product selectivity can be achieved, leading to the synthesis of aniline and its derivatives. These studies not only expand the application scope of electrocatalytic C−N coupling reactions but also provide new green chemical routes for the synthesis of diverse nitrogen‐containing organic compounds.

## 5 Summary and Outlook

In recent years, electrocatalytic C–N coupling reactions have gained significant attention as an efficient and sustainable method for chemical synthesis. This process enables the coupling of nitrogen sources with a variety of carbon sources. The result is the production of a wide range of valuable organonitrogen compounds, including urea, oximes, amino acids, amides, and amines, which are essential in industries ranging from pharmaceuticals to agriculture. One of the major advantages of electrocatalytic C–N coupling is its ability to provide a sustainable and renewable nitrogen sources, thereby reducing reliance on traditional, often environmentally harmful, nitrogen sources like ammonia gas or hazardous reagents. Furthermore, this method operates under milder reaction conditions, eliminating the need for high temperatures or extreme pressures, which are typically required in conventional synthesis processes. As a result, electrocatalytic C–N coupling significantly reduces both energy consumption and the generation of unwanted byproducts, offering a greener alternative to traditional methods. In addition to energy efficiency and environmental benefits, electrocatalytic C–N coupling also demonstrates remarkable selectivity. By carefully controlling reaction parameters such as applied potentials, pH values, and electrolyte composition, it is possible to fine‐tune the reaction pathways, leading to higher yields and fewer side reactions. This selective control is a key advantage when synthesizing complex organonitrogen compounds, which require precise tailoring of the molecular structures for specific applications. Overall, electrocatalytic C–N coupling not only represents a promising avenue for more sustainable and energy‐efficient chemical synthesis but also aligns with broader goals of green chemistry. As advancements in electrocatalyst design and process optimization continue, this method holds significant potential for transforming the way we produce key organonitrogen compounds, supporting a more sustainable and environmentally friendly future for chemical manufacturing.

While electrocatalytic C–N coupling reactions have demonstrated considerable promise in the synthesis of organonitrogen compounds, they still face several significant challenges that need to be addressed to fully realize their potential. These challenges primarily lie in the areas of catalyst design, reaction efficiency, and selectivity. Moving forward, there are several key research directions that could enhance the practicality and scalability of this promising technology.

1. Catalyst optimization and design: One of the most pressing issues in electrocatalytic C–N coupling reactions is the selectivity and stability of the catalysts. Current catalysts often exhibit limited performance under real‐world reaction conditions, leading to issues with efficiency and durability. Future research should focus on the development of catalysts that exhibit both high activity and long‐term stability. Novel catalytic materials, such as dual‐atom catalysts, metal‐organic frameworks, and single‐atom catalysts, show significant promise in improving the efficiency and selectivity of C–N coupling reactions. These materials can offer precise control over active sites, leading to better reaction performance and minimized side reactions. Besides, artificial intelligence (AI) and machine learning (ML), which are increasingly being applied to optimize electrocatalytic processes and design new catalysts. AI and ML have significant potential in accelerating catalyst discovery and optimization by analyzing large datasets and predicting catalyst performance under various conditions. These technologies can efficiently screen chemical spaces, reducing the time and resources needed for experimental trials. For instance, ML algorithms can predict the activity and stability of new catalysts based on the properties of known materials, guiding researchers toward more efficient solutions.AI methods are also being used to optimize reaction conditions, such as applied potential, pH, and electrolyte composition, and reinforcement learning is being explored to improve process efficiency and selectivity in real time. Additionally, AI can correlate experimental data with theoretical models, offering deeper insights into reaction mechanisms and intermediates in electrocatalytic C–N coupling.
2. Enhancing reaction selectivity: In complex multicomponent reactions, achieving high selectivity for the target products remains a substantial challenge. One approach to improving selectivity involves rationally designing the electronic structures of electrocatalysts, which can tailor the reactivity of the active sites to favor specific reaction pathways. Additionally, optimizing reaction conditions such as applied potentials, pH values, and electrolyte composition can help minimize unwanted byproducts, thereby improving the overall selectivity of the reactions. Advanced techniques such as computational modeling and in situ characterizations, can further guide the design of electrocatalysts that exhibit high selectivity and efficiency for C–N coupling.
3. Deeper mechanistic understanding: A deeper mechanistic understanding of electrocatalytic C–N coupling is crucial for advancing this field. Future research should focus on identifying key intermediates, elucidating reaction pathways, and unraveling the associated kinetics by combining advanced in situ characterization techniques—such as in situ infrared spectroscopy, Raman spectroscopy, and synchrotron‐based methods—with theoretical calculations including density functional theory and molecular dynamics simulations. Such comprehensive insights into reaction mechanisms will reveal the interactions between catalyst active sites and reactants, guiding the rational design of highly efficient and selective catalysts to improve C–N coupling efficiency and product selectivity. Moreover, mechanistic studies can help address side reactions and catalyst deactivation issues, thereby facilitating the translation of electrocatalytic C–N coupling toward practical industrial applications. Moving forward, integrating multidisciplinary approaches for systematic mechanistic investigations will provide a solid foundation for achieving efficient, controllable, and green synthesis via electrocatalytic C–N coupling.
4. Valorization of wastes: Another promising area of research lies in the use of electrocatalytic C–N coupling for waste valorization, particularly in converting harmful nitrogen oxides (NO_x_) into valuable organonitrogen compounds. NO_x_, often emitted as pollutants from industrial processes and vehicle exhausts, represent a significant environmental challenge. Electrocatalytic processes that enable the efficient conversion of NO_x_ into useful compounds such as urea or amines offer an innovative solution to both waste reduction and the synthesis of valuable chemicals. This approach not only contributes to environmental sustainability but also presents significant economic opportunities by converting waste products into commercially valuable materials. Scaling this process for industrial applications could have a substantial impact on reducing air pollution and resource consumption.
5. Exploration of industrial applications: Although electrocatalytic C–N coupling reactions have made significant progress in the laboratory, their large‐scale industrial application still faces multiple challenges, including scalability, cost‐effectiveness, selectivity, and yield. To ensure commercial viability, it is crucial to optimize reaction conditions and enhance catalyst performance to improve efficiency and reduce costs. In large‐scale organonitrogen production, key challenges for flow‐cell and MEA electrolyzers include catalyst stability, mass transport, water and electrolyte management, and reactor scalability. Future research should focus on developing durable catalysts, optimizing flow fields and electrode designs to improve mass transport efficiency, improving membrane materials and water management strategies, and scaling up reactors through modular or stacked designs to ensure system efficiency and stability. Furthermore, electrocatalytic reactions in industrial settings also face challenges related to reactor design, mass transport, and electrode stability. To address these issues, research should prioritize optimizing reactor structures, catalyst loading, and electrode configurations to guarantee efficiency and stability during large‐scale operation. Since product separation is typically energy‐intensive, future efforts could explore efficient membrane separation or electrodialysis technologies to reduce energy consumption and costs. Finally, the electricity source for electrocatalytic reactions is critical to their economic viability and environmental impact. Utilizing renewable energy sources such as solar and wind power is key to achieving sustainable development. Research should strengthen the integration and optimization of renewable energy with electrocatalytic processes to improve overall system efficiency and environmental sustainability.
6. Advancement in asymmetric C–N coupling: Another exciting frontier in electrocatalytic C–N coupling is the exploration of asymmetric reactions. Asymmetric C–N coupling reactions, which enable the selective formation of chiral compounds, hold significant promise for the synthesis of complex organonitrogen pharmaceuticals, agrochemicals, and natural products. One approach is the design of chiral molecular electrocatalysts (e.g., chiral metal complexes or organocatalysts) that can coordinate to substrates and impose a well‐defined asymmetric environment during bond formation. Alternatively, chiral‐modified electrode surfaces, where chiral ligands, polymers, or self‐assembled monolayers are anchored to conductive supports, can steer the approach of reactants and favor one enantiomer over the other. Research aimed at optimizing catalyst selectivity for asymmetric transformations, as well as controlling stereochemistry, could lead to groundbreaking advancements in the production of high‐value compounds with specific enantiomeric purity.

Electrocatalytic C–N coupling reactions represent an innovative and sustainable method for the synthesis of organonitrogen compounds. Their potential for reducing environmental pollution and improving energy efficiency positions them as a key technology for green chemistry. As research continues to advance in areas such as catalyst design, reaction optimization, and industrial scalability, electrocatalytic C–N coupling could play a pivotal role in the future of chemical manufacturing. By addressing the current challenges and exploring new avenues such as waste valorization and asymmetric synthesis, this technology has the potential to revolutionize the way we produce nitrogen‐based chemicals and contribute to a more sustainable, environmentally conscious future. With continued progress in both fundamental and applied research, electrocatalytic C–N coupling reactions are poised to become an integral part of the green chemical revolution.

## Conflict of Interest

The authors declare no conflict of interest.
